# High-resolution melting analysis for rapid and cost-effective detection of unnatural base pairs

**DOI:** 10.1016/j.bidere.2026.100090

**Published:** 2026-05-08

**Authors:** Sophie E.J. van der Vlugt, Jelke J. Fros, Vitor A.P. Martins dos Santos, Bauke Albada, Enrique Asin-Garcia

**Affiliations:** a Bioprocess Engineering Group, Wageningen University & Research, 6700 AA, the Netherlands^1^; b Laboratory of Virology, Wageningen University & Research, 6708 PB, the Netherlands^2^; c Laboratory of Organic Chemistry, Wageningen University & Research, 6708 WE, the Netherlands^3^; d LifeGlimmer GmBH, 12163, Berlin, Germany

**Keywords:** Xenobiology, Unnatural base pairs, High-resolution melting analysis, qPCR, SNP detection

## Abstract

Expansion of the genetic alphabet with unnatural base pairs (UBPs) enables new approaches in protein engineering, synthetic biology, and biocontainment. Such applications rely on faithful incorporation and retention of UBPs in semi-synthetic systems, making their detection and monitoring essential tools for xenobiology. Current methods for detecting UBPs in DNA frequently rely on labelling strategies, specialised reagents, or advanced analytical instrumentation. These approaches increase cost, technical complexity, and turnaround time, limiting accessibility and scalability of xenobiological experiments. Here, we establish high-resolution melting (HRM) analysis as a rapid and accessible method for detecting the UBP NaM-TPT3 based on differences in melting temperature of short amplicons. The approach can be directly appended to standard PCR workflows using commonly available qPCR instrumentation, enabling fast and cost-effective screening of UBP incorporation and stability in DNA.

After optimisation of HRM-specific PCR conditions for efficient UBP incorporation, we demonstrate that HRM analysis produces clear and reproducible melting signatures that distinguish natural and unnatural sequences and detect defined variant mixtures. The method is validated using non-amplified DNA controls and LC-MS analysis. By enabling low-cost, workflow-integrated verification of UBP presence using standard laboratory infrastructures, HRM provides a practical screening tool that lowers barriers to experimentation and accelerated iterative development of expanded genetic systems.

## 1 Introduction

Since the discovery of the building blocks that form the blueprint of life, altering and expanding the genetic code has been considered a way to redesign the fundamental principles of living systems. Xenobiology explores how unnatural components can be introduced into biological systems, including reassignment or expansion of codons to enable synthesis of proteins containing non-canonical amino acids, as well as the incorporation of unnatural bases or backbones in DNA or RNA [[1], [2], [3]]. Such alternative biochemistries provide insight into the origins of life and, in being orthogonal to natural life, can provide a novel form of biocontainment based on a “genetic firewall” [4,5]. Though the ethical and philosophical implications of creating semi-synthetic life remain substantial, the field inspires the development of safer and more sustainable biotechnologies with broad societal benefits [[6], [7], [8], [9], [10]].

As the field advances, its molecular toolbox continues to grow. Efforts to expand the genetic alphabet have generated several families of unnatural base pairs (UBPs), typically classified by their pairing mechanism, which may rely on hydrogen bonding or hydrophobic interactions [8]. One of the most extensively studied UBPs to date is NaM-TPT3, developed by the Romesberg group [9,11]. DNA containing this UBP can be amplified by PCR and *in vitro* (reverse) transcribed using several commercial or native polymerases with moderate to high fidelities [[12], [13], [14], [15], [16]]. It has even been incorporated into the *in vivo* environment of *E. coli* and retained for multiple generations [17,18]. However, major challenges remain for the generation of a stable semi-synthetic organism, such as intracellular UNTP availability, the prevention of UBP-loss over time and further optimisation of polymerase incorporation efficiencies and repair mechanisms [1,[18], [19], [20]]. This makes reliable detection of UBP incorporation essential for the development of xenobiological systems.

Several methods have been developed to detect UBPs in nucleic acids by exploiting their chemical differences from natural bases. Sanger sequencing of UBPs, originally developed for the Ds-Pa pair (Hirao group), causes a dead-end stop of the reaction at an UB location in the absence of complementary (dd)UBs, and can even be used quantitatively [21,22]. This protocol, however, is typically performed in-house, requiring optimisation and specialised equipment and expertise. More recent variations of Sanger such as gap sequencing or bridge-base approaches rely on the controlled conversion of an unnatural to a natural base pair by introducing additional modified (unnatural) nucleotides in the reaction that facilitate detection, or employing a multi-step stalling and primer extension approach relying on unnatural-natural mispairing [[23], [24], [25]]. Similarly, the Romesberg group developed a gel-shift assay that uses a biotin-labelled dNaM, in which the interaction with streptavidin causes UBP-containing DNA to migrate slower on an agarose gel compared to natural DNA [12]. While the use of modified or labelled nucleotides presents valuable tools for site-specific identification and visualisation of UBPs in genetic material, they require additional synthesis and amplification steps, complicating their use [[26], [27], [28]]. A more precise, yet costly and destructive detection method is liquid chromatography-mass spectrometry (LC-MS), which separates small DNA fragments or digested nucleosides and measures their exact masses [14,19]. LC-MS can sensitively detect small fractions of base changes in the 10s of Daltons range, but is mainly qualitative and requires considerable amounts of input material [14,29]. Lastly, nanopore sequencing has also been explored for UBP detection, but is not yet widely available. While a proof-of-concept was recently achieved for Ds-Px using standard ONT platforms, nanopore sequencing of other UBPs still relies on more specialised systems and is hampered by the development of base calling algorithms with sufficient accuracy and training data [[30], [31], [32]].

While these approaches are technically powerful, they differ in speed, accessibility, and cost. Additional processing or amplification steps increase the risk of artefacts, and the need for specialised reagents, instrumentation, or expertise limits routine use in standard molecular biology laboratories. For applications that require frequent or large-scale screening, such as studies focused on genetic stability, optimisation of polymerase performance, or biocontainment strategies, these constraints significantly slow experimental progress and increase cost. This hampers the accessibility of xenobiological research and the development of both experimental toolboxes and future applications.

Here, we propose high-resolution melting (HRM) analysis as a rapid, accessible, and cost-effective method for detecting UBPs in PCR products. HRM analysis is based on sequence-dependent differences in DNA melting behaviour: G-C base pairs, which form three hydrogen bonds, exhibit higher melting temperatures (Tm) than A-T pairs. Small differences in amplicon length or GC content can therefore be detected by monitoring fluorescence loss of a saturating DNA-binding bye during controlled heating in standard qPCR instruments [33,34]. HRM enables discrimination of single-nucleotide variants and mixed populations relative to a reference sequence with high sensitivity [35]. Originally developed for mutation scanning and SNP genotyping, HRM is now widely used for epigenetic screening, food analysis and authentication, species identification and monitoring (plants, microbes), and molecular diagnostics for pathogen screening and detection [[36], [37], [38], [39]]. Commercial kits and software further facilitate routine implementation as a non-destructive and inexpensive analytical technique.

**Fig. 1:**
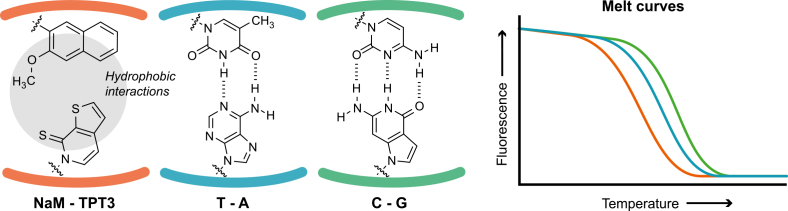
**Principle of UBP detection by HRM.** The unnatural base pair (UBP) NaM-TPT3 forms no hydrogen bonds, in contrast to the natural T-A (two hydrogen bonds), and C-G (three hydrogen bonds) pairs. This influences their melting behaviour, as more hydrogen bonds require more energy to break the dsDNA structure and therefore cause a higher melting temperature. These small differences can be visualised in high resolution melting (HRM) analysis, by measuring the melt curve of a short dsDNA fragment using a fluorescent dsDNA-binding dye in a qPCR machine. We hypothesize that the melt curve of a UBP-containing fragment shifts left compared to natural sequences, enabling detection via HRM analysis.

We hypothesize that replacing hydrogen-bonded base pairing with hydrophobic interactions in the NaM-TPT3 UBP results in a reduced Tm relative to equivalent natural sequences (Fig. 1). Here, we develop HRM acquisition and analysis protocols for detecting NaM-TPT3 in amplified DNA. We demonstrate that UBP incorporation produces reproducible and quantifiable changes in melting behaviour, including in mixed populations. Results are independently validated by LC-MS and nanopore sequencing. Together, this work establishes HRM as a simple and scalable method for monitoring UBPs, substantially lowering technical and financial barriers to experimental studies of expanded genetic systems.

## 2 Material and methods

### 2.1 Reaction composition and cycling conditions

Template DNA was generated by annealing two complementary single-stranded oligonucleotides (Table S1, stock concentrations 10 mM). Equimolar mixtures were heated in Milli-Q (MQ) water to 95 °C for 5 min in a heat block and allowed to slowly cool down to room temperature. dsDNA formation was verified by agarose gel electrophoresis, and concentrations determined using a Nanodrop spectrophotometer. Templates were subsequently diluted in MQ water to approximately 10 pg/μL for PCR templates and 500 ng/μL for positive controls.

PCR reactions were assembled with the following final concentrations in MQ water: OneTaq standard reactions buffer (20 mM Tris-HCl, 22 mM NH4Cl, 22 mM KCl, 1.8 mM MgCl_2_, 0.06% IGEPAL® CA-630, 0.05% Tween® 20) (*New England Biolabs (NEB)*), 400 μM of each canonical dNTP and, where indicated, 200 μM dNaMTP and dTPT3TP, an additional 2 mM MgCl_2_, 0.8 μM forward and reverse primers (Table S1), 0.1 pg/μL template DNA, 0.025 U/μL OneTaq DNA polymerase (*NEB*), and 1x LCGreen PLUS dye (supplied as 10X solution in 10 mM Tris-HCl, pH 7.4, 0.1 mM EDTA) (*BioFire Defence*). For each HRM experiment, reactions were assembled at 35 μL scale and subsequently aliquoted into three 10 μL reactions. PCR was performed in 10 μL volumes using a CFX96 Touch Real-Time PCR Detection System on a C1000 Touch Thermal Cycler (*Bio-Rad*), with an average ramp rate of 3.3 °C/s. The cycling protocol consisted of an initial denaturation step at 94 °C for 2 min, followed by 30 cycles of denaturation at 94 °C for 30 s, annealing at 59 °C for 40 s, and elongation at 68 °C for 1 min 30 s, and a final extension at 68 °C for 3 min. Immediately thereafter, melt curves were acquired by cooling to 50 °C and increasing the temperature in 0.1 °C increments with a 10 s hold at each step, with fluorescence measured (excitation 450-490 nm, emission 515-530 nm) up to 95 °C.

Amplification and melting data were acquired using CFX Maestro Software (*Bio-Rad*). Amplification curves are reported as relative fluorescence units (RFU) per cycle, melt curves as RFU per temperature, and melt-peak curves as the negative derivative of fluorescence over temperature (-dF/dT) per temperature. After PCR and melt-curve acquisition, amplicons were analysed by agarose gel electrophoresis (using EtBr staining) and purified using the NucleoSpin® Gel and PCR Clean-Up Kit (*Macherey-Nagel*) according to the manufacturer's protocol.

Positive control reactions were assembled using the same reaction composition, except that the template DNA was added at 75 ng/μL and no OneTaq polymerase was included. As no amplification was performed, 10 μL reactions were assembled and directly subjected to a 3 min equilibration step at 68 °C prior to melt-curve acquisition as described above.

For mixtures experiments, PCR reactions were assembled at 60 μL scale per replicate. Of each reaction, 10 μL was analysed in the qPCR instrument to determine amplification efficiency (up to the 68 °C hold step), while the remaining 50 μL was amplified in a conventional thermal cycler using the same cycling protocol. Amplified products were subsequently mixed volumetrically in defined rations (10 μL total volume per condition) directly in qPCR plates. Only the melt-curve acquisition protocol was performed for these samples, analogous to positive control samples.

### 2.2 Analysis of results

Raw data were exported from the CFX Maestro Software (*Bio-Rad*) and HRM analysis was performed using Microsoft Excel. For each experiment, three technical replicates were averaged to generate mean melt curves (RFU vs. temperature). Pre- and post-melt baseline temperatures were defined at 83 °C and 90 °C for all experiments.

For each sequence variant, the mean post-melt fluorescence was subtracted from all mean RFU values, and fluorescence was normalised to the percentage between the pre- and post-melting baselines at each acquisition temperature. Difference curves were generating by subtracting the normalised fluorescence values of the reference sample from those of each variant within the same experiment. Relative melting differences were plotted over the 83-90 °C temperature range. The average normalised fluorescence values and differences per experiment (mean of three technical replicates per experiment) were visualised using GraphPad Prism (*GraphPad*) by plotting the mean values across experiments. Standard errors of the mean were calculated and displayed using Prism.

Melt-peak temperatures were determined per technical replicate by the CFX Maestro Software as the temperature at which the -dF/dT vs temperature curve reached its maximum (one value per replicate). For each condition, replicate values were averaged across three independent experiments, and standard errors of the mean values were calculated in Prism. Statistical significance for mixture discrimination (Fig. 5B and D and Table S2) was assessed using a one-way ANOVA with multiple comparisons in Prism. Data were analysed under the assumptions of normal distribution and equal homogeneity of variance.

### 2.3 Nanopore sequencing

Nanopore sequencing was performed commercially (Plasmidsaurus Premium PCR Amplicon Sequencing Service). Purified PCR products were diluted according to the provider's instructions and submitted for sequencing. Sample quality and integrity were externally verified by assessment of average read lengths per sample (Fig. S3). Per-base read data (base identity, mismatches, deletions, insertions) were analysed in Microsoft Excel by calculating percentages relative to total reads per sample. Mean values of two replicates were calculated and plotted (Fig. S3).

### 2.4 LC-MS analysis

Samples for LC-MS analysis were prepared by restriction digestion and manual purification of PCR amplicons and positive controls. Following melt-curve acquisition, technical replicates for each variant were pooled, and 10-15 units of restriction enzyme *Hae*III (*NEB*) was added directly to the reactions (OneTaq reaction buffer supports cleavage activity). Reactions were incubated overnight at 37 °C, after which complete digestion was verified on a 2% agarose gel.

Because digestion yielded fragments of 46 and 74 bp, standard column-based purification was not feasible. DNA content was therefore purified by phenol-chloroform extraction followed by ethanol precipitation. To this end, each reaction was adjusted to 200 μL with MQ water. Subsequently, 10 μL of 10% SDS and 5 μL proteinase K (20 mg/mL) were added, and mixtures were incubated at 37 °C for 20 min. An equal volume of phenol-chloroform-isoamyl alcohol was added, followed by vortexing and centrifugation for 10 min (14.000 rpm at 4 °C). The aqueous phase (∼200 μL) was transferred to a fresh tube, supplemented with 1 μL glycogen, 20 μL 3M NaAc, and 500 μL (∼2 vol) 100% ethanol, and incubated overnight at −20 °C for precipitation. DNA was pelleted by centrifugation (15 min, 14.000 rpm, 4 °C), washed twice with 1 volume of 70% ethanol, air-dried at 60 °C, and resuspended in MQ water. DNA integrity was confirmed by 2% agarose gel electrophoresis and concentrations were determined with spectrophotometry.

HPLC-ESI-MS was performed using an Agilent 1290 Infinity UHPLC system coupled to a Q-Exactive Focus Mass Spectrometer (using Xcalibur data acquisition Software (*Thermo Scientific*)). Preceding and following measurements, the system was cleaned with 100% methanol and calibrated according to the manufacturer's instructions.

Approximately 30 pmol of purified DNA was injected for HPLC separation onto a DNAPac RP column (4 μm, 2.1 × 100 mm, *Thermo Scientific*) at 60 °C. Solvent A consisted of 10 mM triethylammonium acetate (TEAA) in MQ water; solvent B consisted of 10 mM TEAA in MQ water/acetonitrile (75:25). The following 25 min protocol was applied: 15% B for 5 min followed by a gradient from 15 to 70% B in 7 min at a flow rate of 0.3 mL/min. Subsequently, B was increased to 95% over 3 min and washed during 5 min, after which the starting gradient of 15% B was restored and the following sample injected.

Spectra were recorded at DAD detector wavelengths of 215, 230, 260, and 360 nm in the HPLC and 260 nm in the MS. Mass acquisitions were performed in negative mode and a 500-3000 *m*/*z* range.

HPLC spectra were exported using OpenLab CDS Software (*Agilent*), and MS data analysed using Xcalibur Freestyle Software (*Thermo Scientific*) by integrating individual peaks. Mass spectra were deconvoluted and plotted using UniDec Software (settings: full range, charge range 1-30, mass range: 5000-40000 Da, sample every 1 Da, peak detection every 1 Da, threshold 0.1 or 0.2) [40].

## 3 Results

### 3.1 Development and verification of an HRM protocol suitable for NaM-TPT3 amplification

**Fig. 2:**
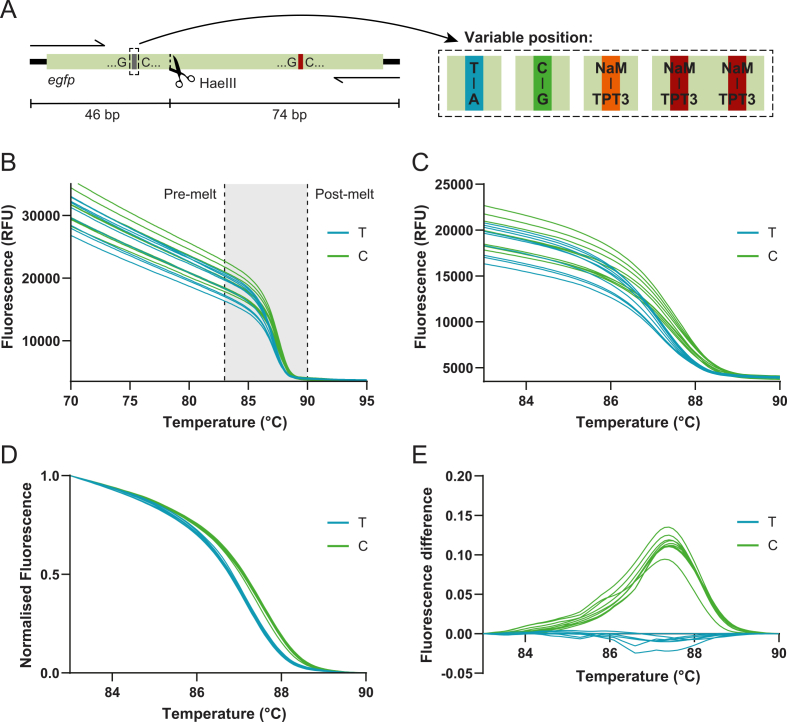
**Procedure of HRM analysis and differentiation between two double-stranded DNA molecules containing a single-nucleotide variant, i.e., a T-A (denoted ‘T’) or C-G base pair (denoted ‘C’)**. (**A**) Schematic representation of PCR templates and amplicons, comprising a fragment of the *egfp* sequence with a single-base variant at position 38, either natural bases T-A (blue) or C-G (green), a single NaM-TPT3 substitution (orange) or two NaM-TPT3 substitutions at positions 38 and 90 (red). Further indicated are the PCR primers, the sequence context of the variable sites (GXC) and a naturally occurring enzymatic restriction site (HaeIII). **(B)** Raw melt curves from three independent experiments, each with three technical replicates. Pre- and post-melt temperatures were set at 83 and 90 °C. **(C)** Zoom of the melting region of variants T and C shown in panel B. **(D)** Corresponding per-sample normalised melt curves of all replicates. **(E)** Difference plots obtained by subtracting the ‘C’ curves with the reference ‘T’ curve (one reference per experiment). Also see Fig. S1.

To enable amplification of UBP-containing DNA and subsequent detection of UBPs in the amplicon, we first adapted PCR and HRM protocols to the use of OneTaq polymerase, which is preferred for the incorporation of NaM-TPT3 [12,41]. Increased salt concentrations can enhance both mutation discrimination and dUNTP incorporation; therefore, MgCl_2_ was supplemented to a final concentration of 3.5 mM [19,34,35,42,43]. To ensure sufficient amplification and resolution, primers were designed following standard guidelines and validated for specificity, and amplicon length kept short (<300 bp is generally recommended). In this study, templates of 120 bp were commercially synthesised and HPLC purified. The UBP-containing template contained UB 5SICS instead of TPT3 in the reverse strand, as the phosphoramidite of the latter is currently not commercially available, but is easily replaced by TPT3 upon PCR amplification [44,45]. A fragment of *egfp* was amplified, which included a single variable base pair at position 38, a previously identified permissive site for NaM-TPT3, and a second UBP site at position 90 [17,46]. Although sequence context of the NaM-TPT3 matters most in *in vivo* replication and translation, the most stable and widely used context GXC was used in this study [31,41,47,48] (Fig. 2A). Lastly, the HRM-optimised dye LCGreen Plus was included to ensure sensitivity of melt-curve acquisition.

PCRs were performed in 10 μL volumes on a standard qPCR instrument (Bio-Rad CFX96 Real-Time System). The amplification protocol followed the manufacturer's recommendations for OneTaq, with two important adaptations. First, UB-PCR protocols typically use a maximum of 30 amplification cycles to minimise misincorporation of natural bases at UBP positions [22,26]. However, to reliably compare melt curves across samples, amplification ideally proceeds to completion, with threshold cycle (Ct) values falling within a narrow (three-cycle) range. Achieving this requires careful balancing of template input; with template identical to the amplicon, 10 pg per reaction was found to be sufficient. Second, UB-PCRs employ significantly longer extension times than standard reactions (for OneTaq, 1 kb/min) to ensure efficient UBP incorporation [42,49]. Both conditions were consistently applied throughout this work for both natural and unnatural PCRs, to maintain comparability. Immediately after amplification, melt curves from 50 to 95 °C were acquired with 0.1 °C temperature increments every 10 s to allow complete equilibration.

To assess the design and sensitivity of the HRM protocol, we first tested whether a single natural base-pair change at the variable position, either T-A (hereafter ‘T’) or C-G (hereafter ‘C’), could be reliably distinguished (Fig. 2A). PCR amplification and melt-curve acquisition were performed in three independent experiments, each comprising three technical replicates. All reactions reached completion, yielding Ct values between 11 and 13 (Fig. S1). Subsequent HRM analysis was performed on the resulting melt curve data (Fig. 2). From the raw curves (Fig. 2B), pre- and post-melting baselines were defined at 83 and 90 °C, respectively, to normalise sample-to-sample variation (Fig. 2C). Per-sample normalised fluorescence values (Fig. 2D) were then used to generate difference curves by selecting one replicate of T per experiment as the reference and plotting the deviation of all remaining samples relative to this reference (Fig. 2E). By subtracting a reference curve from each sample, difference plots suppress shared background features and accentuate subtle, sequence-dependent shifts in melting behaviour that are otherwise difficult to resolve [34]. We observed a clear and reproducible separation between the T and C variants, with a positive difference curve of C representing a higher melting temperature of the sample. A single base pair substitution produced an approximately 0.4 °C shift in melt-peak temperature (Fig. S1), similar to previous reports using standard HRM designs [50]. This demonstrates that the UBP-adapted HRM protocol reliably detects a single natural base-pair substitution in a 120-bp amplicon.

### 3.2 Detection of UBP NaM-TPT3 with HRM analysis

We next applied our HRM method to distinguish natural DNA sequences from those containing the UBP NaM-TPT3. PCR amplification and melt-curve acquisition were performed as described above on templates containing the T-A, C-G, or NaM-TPT3 variants (hereafter denoted ‘T’, ‘C’, and ‘NaM’). We additionally included a template containing two NaM-TPT3 pairs (denoted ‘2NaM’), taking into account the same sequence context (GXC) and sufficient spacing between two UBPs [24,47,48,51] (Fig. 2A). All PCR reactions of single-base variants reached completion with Ct values of 15-16. Gel electrophoresis confirmed comparable product concentrations and the absence of substantial truncations (>10 bp) for all the single-base variants, including NaM. In contrast, the 2NaM variant showed reduced amplification efficiency (Ct 19-21) and product formation, as observed on a 2% agarose gel (Fig. S2).

**Fig. 3:**
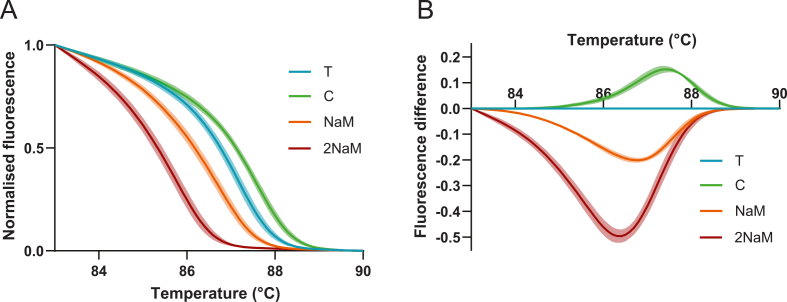
**HRM analysis of amplicons containing single natural or single or double unnatural base pair variations. (A)** Average normalised melt curves of four sequence variants T, C, NaM and 2NaM (three independent experiments, each with three technical replicates). **(B)** Corresponding difference plots relative to reference sample T, showing a progressive reduction in melt temperature for amplicons containing one or two UBPs compared to both natural sequences. Also see Fig. S2.

Normalised melt curves showed that samples containing T and C displayed melting behaviour comparable to that observed previously (Fig. 2E), whereas UBP-containing samples exhibited negative shifts in melt curves, signifying reduced melting temperatures (Fig. 3A). Corresponding difference plots accentuated these differences, with the NaM amplicon exhibiting a clearly negative curve relative to reference T, which became more pronounced for the 2NaM product (Fig. 3B). Melt-peak temperatures show clear 0.5-1.4 °C reductions corresponding to the presence of one or more UBP (Fig. S2). However, it should be noted that the reduced amplification efficiency of the 2NaM samples reduces the quantitative accuracy of its melt profile. Together, these results demonstrate that the presence of one or more UBPs in a short PCR amplicon causes a measurable reduction in melting temperature that can be reliably detected by HRM analysis.

### 3.3 Accuracy of UBP-detection

**Fig. 4:**
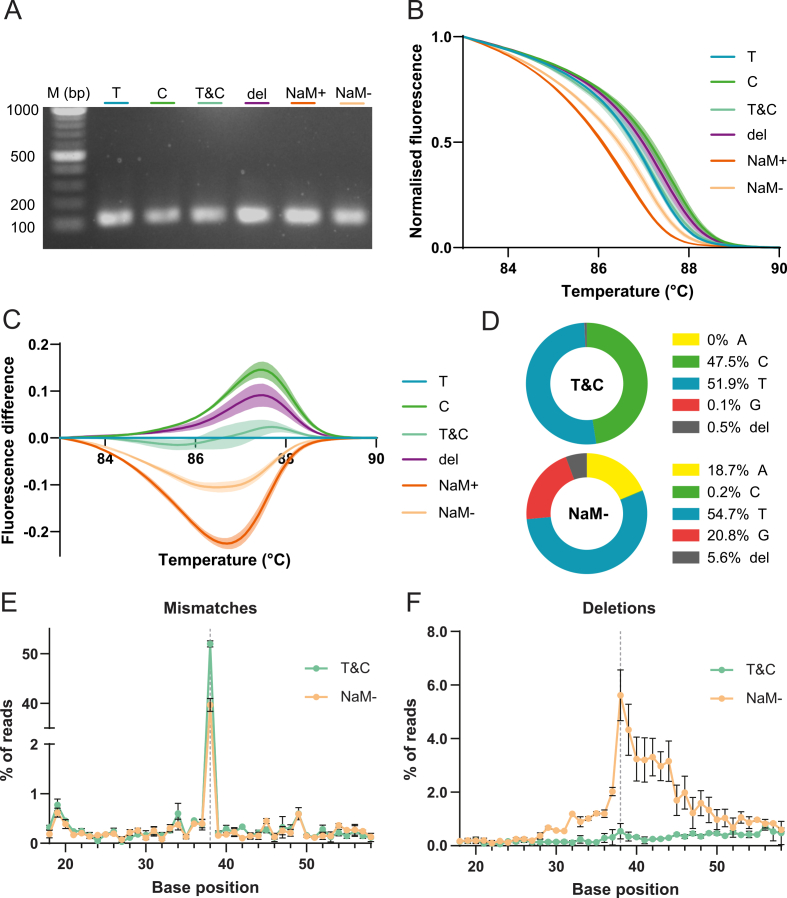
**Detection and discrimination of natural and unnatural sequence variants by HRM and nanopore sequencing.** PCR templates containing either T-A, C-G, NaM-TPT3, or a single-base deletion at a defined variable position were amplified and analysed by HRM. Samples T&C and NaM- were subsequently sequenced by nanopore. **(A)** Gel electrophoresis of one replicate of amplicons used for HRM analysis. **(B)** Average normalised melt curves from three experiments with three technical replicates each. **(C)** Corresponding average difference curves relative to reference T. **(D)** Base identity distribution at the variable position of both samples as percentage of all reads. **(E)** Percentage of reads with a base identity differing from reference T, based on two sequencing replicates; the variable position (38) is indicated by a grey dashed line. **(F)** Percentage of reads containing deletions relative to reference T, based on two sequencing replicates; the variable position (38) is indicated by a grey dashed line. Also see Fig. S3.

To investigate the accuracy of the HRM protocol, we next examined how potential loss of the UBP during PCR amplification affects melting behaviour. To this end, PCRs were performed either in the presence of unnatural dNTPs (dUNTPs; NaM+) or in their absence (NaM-). In both cases, amplification of the UBP-containing template displayed no visible reduction in product formation and only slightly higher Ct-values (Fig. 4A and S3), suggesting that OneTaq efficiently converts a UBP-containing template into fully natural sequence(s) in the absence of dUNTPs. We hypothesised that the polymerase introduces a mutation or deletion at the UBP locus, generating a heterologous population of amplicons.

To account for and compare these possibilities, two additional sequence variants were included: one containing a mixture of T and C templates (‘T&C’), and one containing a template with a single base pair deletion at the variable position (‘del’, yielding a 119-bp amplicon). PCRs were performed using the same protocol as above and all reached comparable Ct values (14-17) and product yield and length, as confirmed by 2% agarose gel analysis (Fig. 4A and S3). The mixed-template sample T&C displayed melting behaviour similar to the pure T sample (see next section), whereas the deletion variant melted at a higher temperature than the 120-bp T amplicon, likely due to increased GC content (Fig. 4B–C and S3).

The resulting NaM- amplicons exhibited lower melting temperatures than all natural variants, resulting in a negative difference curve relative to reference T, while melting temperatures were clearly higher than for NaM+ (Fig. S3). Although the NaM- amplicon must be of natural composition, its melt signature does not resemble any of the other natural variants analysed, which all display positive difference curves relative to T (Fig. 4C). To clarify its sequence identity, nanopore sequencing was performed on NaM- amplicons, with the natural T&C mixture included as a control. Fig. 4E displays the percentage of mismatches relative to a reference sequence containing T at the variable position and 20 bp of flanking sequence on either side. As expected, mixture T&C exhibited approximately 50% identity to the reference T, with the remaining reads corresponding to the C template (Fig. 4D). For NaM-, approximately 55% or reads matched T, whereas ∼21%, and ∼19% of reads were identified as G and A, respectively, at the variable position. This indicates that natural bases T, G, and A readily replaced NaM during amplification by OneTaq, whereas replacement by C is barely observed (Fig. 4D). Additionally, NaM- contained a notably higher fraction of deletions (5,7%) at the variable position compared with the natural T&C control (0,5%), while insertions remained at background levels for all samples (Fig. 4F and Fig. S3). Deletions also occurred more frequently downstream of the variable site, suggesting that OneTaq may introduce multi-nucleotide deletions when unable to incorporate the corresponding dUNTP, which likely contributes to the observed melt profile but would be hard to observe on gel (Fig. 4A–C). The slight broadening of the melt peak in the raw melt peak data supports this interpretation (Fig. S3). Further work will be required to elucidate the molecular mechanisms underlying polymerase errors at UBP sites in the absence of dUNTPs.

Collectively, these results show that multiple sequence variants can be reliably distinguished using the UBP-adapted HRM protocol, but that mixed amplicon populations produce ambiguous melting profiles and that OneTaq can introduce single or multiple mutations at UBP positions when dUNTPs are absent.

### 3.4 Identifying and quantifying amplicon mixtures

**Fig. 5:**
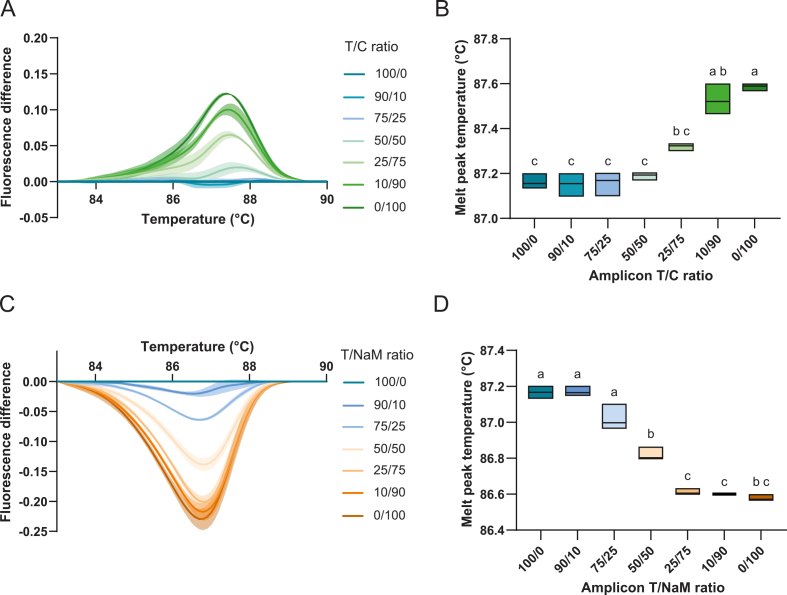
**Identification of variant mixtures by HRM. (A)** Average difference curves of T/C amplicon mixtures from three independent experiments with three technical replicates each. Following PCR, T and C amplicons were mixed in defined volume ratios and analysed by HRM. **(B)** Melt-peak temperatures of T/C amplicon mixtures corresponding to panel A based on fluorescence. Groups that share the same letter are not significantly different, whereas groups with different letters differ significantly (p < 0.05). Details on statistics in Table S2. **(C)** Average difference curves T/NaM+ amplicon mixtures from three independent experiments with three technical replicates each. **(D)** Melt-peak temperatures of T/NaM+ amplicon mixtures based on fluorescence corresponding to panel B. Groups that share the same letter are not significantly different, whereas groups with different letters differ significantly (p < 0.05). Details on statistics in Table S2. Also see Fig. S4.

To assess the sensitivity of the method, we next tested whether mixtures of sequence variants could be distinguished and potentially quantified. Amplicons of variants T, C, and NaM+ of similar quantities, based on endpoint fluorescence, were mixed in defined volume ratios (0, 10, 25, 50, 75, and 100%) and analysed immediately by HRM (Fig. S4). The resulting difference curves relative to reference T revealed a nonlinear shift in melting behaviour across three independent experiments (Fig. 5).

For mixtures of T and C, clear changes were observed relative to the 100% T reference when the proportion of variant C increased from 50% to 100%. However, below 50% C, melting behaviour no longer differed distinguishably from the reference (Fig. 5A). This asymmetry is also reflected in melt-peak temperatures: samples containing 0-50% C consistently exhibited peaks around 87.1 °C, whereas mixtures containing 50-100% C showed significantly higher peaks, reaching 87.6 °C at 100% C (Fig. 5B). These results indicate that HRM can discriminate T/C amplicon mixtures when the majority of the sample contains a C-G base pair, but loses resolving power when its fraction drops below 50%.

A similar, but mirrored, pattern was observed in mixtures of T and NaM+ amplicons. Decreasing the proportion of variant T from 100% to 25% relative to NaM resulted in clear changes in melting behaviour (Fig. 5C). However, above 75% NaM relative to T, further changes in composition no longer produced distinguishable melting differences compared with the pure NaM+ amplicon. This behaviour was also reflected in melt-peak temperatures, which could only be significantly distinguished between a T/NaM ratios of approximately 75/25% and 25/75% (Fig. 5D). These results indicate that loss of NaM incorporation in more than ∼25% of amplicons can be reliably detected using HRM analysis.

### 3.5 Validation of the method with positive controls and LC-MS

Given that OneTaq can incorporate natural bases in place of a UBP in the absence of corresponding dUNTPs, we designed two complementary experiments to determine whether the HRM analyses indeed reflect reliable PCR amplification of UBP-containing templates. To validate the previously obtained melt profiles and difference curves independently of PCR, we performed HRM analyses directly on (UBP-containing) DNA of Fig. 4, Fig. 5A (T, C, T&C, del, NaM and T/C mixtures) while omitting the OneTaq amplification step. A large amount of dsDNA template (750 ng) was generated by annealing complementary single-stranded oligonucleotides and subjected directly to melt-curve acquisition and HRM using the same reaction composition. This format allowed us to investigate the effect of mismatches on melting behaviour, so a T/G mismatch was included by annealing top and bottom strands of sample T and C, respectively.

**Fig. 6:**
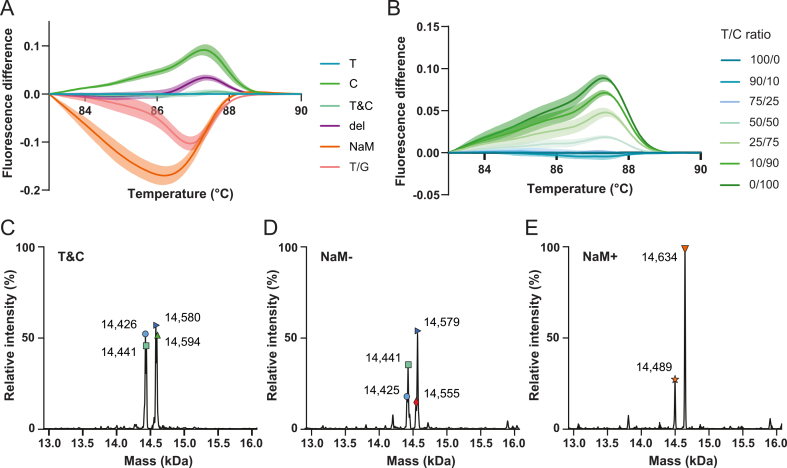
**Positive controls and LC-MS validation of HRM results. (A and B)** Average difference curves generated from melt profiles of high-quantity template DNA analysed without PCR amplification. Data from three independent experiments with three technical replicates each. **(A)** Difference curves for positive control samples T, C, T&C, del, NaM and mismatch T/G (see Fig. 4C). **(B)** Difference curves for mixtures of positive control samples T and C (see Fig. 5A). Also see Fig. S5. **(C-E)** Deconvoluted MS spectra obtained from ESI-MS analysis of digested and purified HRM samples in Fig. 4C. **(C)** Zoom of deconvoluted –MS (negative mode) of T&C. Calculated masses: M (T(sense) + dG) = 14,441 Da, M (T(sense) + dA) = 14,425 Da, M (T(antisense) + dA) = 14,579 Da, M (C(sense) + dG) = 14,425, M (C(antisense) + dA) = 14,594 Da. **(D)** Zoom of deconvoluted –MS (negative mode) of NaM-. Calculated masses: M (T(sense) + dA) = 14,425 Da, M (T(sense) + dG) = 14,441 Da, M (T(antisense) + dA) = 14,579, M (T(antisense) + dC) = 14,555 Da, M (G(antisense) + dA) = 14,555 Da. **(E)** Zoom of deconvoluted –MS (negative mode) of NaM+. Calculated masses: M (NaM(sense) + dTPT3) = 14,489 Da, M (NaM(antisense) + dNaM) = 14,634 Da. See Table S3 and Figs. S6–8 for all spectra and expected and observed masses.

The resulting difference curves showed the same overall HRM profiles as those obtained after PCR amplification, although with smaller absolute fluorescence differences between samples (Fig. 6A–B). The T/G mismatch showed a negative difference curve, though less pronounced as the UBP. A leftward broadening of the difference curves was observed for all samples, originating in corresponding shifts melt peak temperatures (Fig. S5). This effect is likely caused by imperfect equimolar annealing of the ssDNA oligos, which generates a distribution of heteroduplex species and consequently broadens (and flattens) the melting transition. The NaM difference curve in particular displayed pronounced broadening, matching low quality raw melt data (Fig. S5). This is reflected in increased variation across replicates and prevented reliable determination of melt-peak temperatures, thereby limiting assessment of PCR-product purity based solely on peak positions. The reduced data quality was not caused by insufficient input material or template integrity, as confirmed by gel electrophoresis (Fig. S5). Instead, it is likely caused by the presence of d5SICS instead of dTPT3 in the template strand, whose slightly altered chemical properties would exhibit a different melting behaviour.

Nevertheless, the same relative trends and mixture-dependent differences seen in Fig. 4, Fig. 5B were reproduced in these experiments, confirming that OneTaq amplification followed by the HRM workflow robustly detects single base-pair changes in short DNA fragments and can distinguish certain variant mixtures, albeit with limited quantitative resolution.

Lastly, we employed LC-MS to validate our HRM results [14,42]. Measuring the exact masses of PCR amplicons corresponding to the HRM profiles confirms sequence identity at the variable base position. To resolve mass differences in the 10-50 Da range, restriction digestion was performed directly on PCR products following melt-curve acquisition, reducing the UBP-containing fragment to 46 bp (approximately 14 kDa per ssDNA strand) (Fig. 2A). Digested products were separated by reversed-phase HPLC and analysed by ESI-MS, and molecular masses were calculated by spectra deconvolution. For the amplicons shown in Fig. 4C and the annealed DNA in Fig. 5A, LC-MS was performed on one replicate (Figs. S6–8). Each amplicon fragment generated three expected masses: one corresponding to the sense strand and two corresponding to the antisense strand, as OneTaq is a mixture of DeepVent and Taq polymerases, the latter introducing a 3’ adenine overhang [52] (for unamplified DNA, two masses were expected) (Table S3). Although not all expected masses were observed in every spectrum, all obtained deconvoluted masses could be assigned to the predicted species.

For samples T&C and NaM-, both of which displayed a chromatographic peak of equal intensity at ∼7.8 min, the obtained masses corresponded to T and C sequences for T&C, and to T and G sequences for NaM- (Fig. 6C–D and Figs. S6–8 an**d Tabel S3**). These mass distributions are consistent with the nanopore sequencing results shown in Fig. 4D, although the A substitution observed by sequencing for NaM-was not detected by LC-MS. For all natural samples, a consistent mass addition pattern to the expected masses was observed, adding + dG to the sense strand and +dA or + dC to the antisense strand. These additions are likely attributable to residual polymerase activity during overnight digestion (see discussion and Table S3). Interestingly, samples containing UBPs exhibited the same addition pattern, while also showing masses consistent with additions of both dUNTPs. For NaM+, deconvoluted masses of two dominant double UBP-containing species were obtained at ∼8.9 min, confirming successful UBP incorporation (Fig. 6E). Several secondary peaks at ∼8.3 min contained species that displayed the natural (+dG, +dA/dC) addition patterns and most likely also incorporated the UBP at the variable position (Figs. S6–8 and Table S3). No truncated fragments at the UBP position were detected.

Together, these results confirm that NaM-TPT3 is efficiently incorporated during amplification in the presence of corresponding dUNTPs, and that the observed HRM melt and difference curves reflect the presence of UBPs in the amplicons relative to natural sequences. Although no evidence of natural base misincorporation was detected for NaM+, the distribution of species over several LC-MS peaks does not allow conclusive quantitative assessment of product purity.

## 4 Discussion

In this work, we propose high-resolution melting (HRM) analysis as a method for detecting unnatural base pairs (UBPs) in short PCR amplicons and dsDNA fragments. We demonstrate that HRM reliably distinguishes the UBP NaM-TPT3 from natural base variants using standard qPCR instrumentation available in most molecular biology laboratories. Together with its speed and ease of use, this makes the method more accessible than previously developed UBP detection approaches, which often rely on additional reagents or specialised instrumentation. Because HRM analysis is non-destructive, PCR products remain available for downstream purification and analysis, enabling HRM to function as an internal verification step within genetic engineering workflows. Overall, HRM provides a rapid and cost-effective approach for UBP detection, lowering technical and financial barriers for studies investigating the stability and functionality of expanded genetic systems.

While several commercial HRM kits exist, the use of UBPs in PCR required adaptations of reaction composition and cycling conditions to ensure efficient UBP incorporation [12,14]. Under these optimised conditions, our protocol reliably discriminated single-base sequence variants and detected defined mixtures between two natural base pairs. We further show that incorporation of a single UBP leads to a measurable shift in melting behaviour and a negative HRM difference curve relative to samples of natural sequence. A UBP incorporation efficiency of at least 25% significantly altered melting behaviour, although the quantitative power for discriminating mixtures remained limited, and melting temperatures changed only modestly above a 75% NaM/T ratio. Amplification of templates containing a single UBP proceeded with near-equal efficiency relative to natural-sequence templates, and product yield and melt-peak intensity were not substantially reduced. In contrast, when two UBPs were present in the template, even when not in direct proximity to each other, both amplification efficiency and melt-peak intensity decreased notably (Figs. S2–3). As a result, the 2NaM profiles were less reliable in an inherently comparative method such as HRM analysis, impeding its use for detecting multiple UBPs for now. Reduced product formation also precluded LC-MS analysis, as measurements fell below the limit of detection.

Although HRM was sensitive and consistent across replicates, the quantitative resolving power is lower than that of previously developed UBP detection methods such as in-house Sanger sequencing or gel-shift assays, which have reported greater quantitative capability for assessing sample purity [12,22]. Likewise, determination of exact sequence identity is better achieved with methods such as nanopore sequencing, LC-MS, or the bridge-base approach, although these techniques are often more costly or resource-intensive [14,24,31,53]. The most suitable UBP detection strategy therefore depends on the specific experiment and intended application. We position HRM primarily as a rapid, low-effort screening tool: melt-curve acquisition can be appended to an existing PCR workflow with minimal adaptations beyond inclusion of a saturating dye, enabling time- and resource-efficient monitoring while remaining compatible with downstream analyses by additional detection methods when higher resolution is required. In this context, our method is well suited for rapid tracking of UBP retention during genetic engineering workflows and for larger-scale screening efforts, such as those needed for future biocontainment applications.

Despite the fact that our study did not detect impurities in UBP amplification and reported polymerase fidelities are high, validating the purity of the NaM+ amplicon remains challenging. To exclude potential biases and misincorporations arising from PCR amplification, HRM analysis was conducted directly on large quantities of annealed dsDNA templates as positive controls. These controls confirmed the overall trends observed after PCR and even seem to be able to discriminate between UBPs and mispaired bases. However, lower fluorescence values resulted in broadening and reduced intensity of the difference curves, precluding strong conclusions regarding low-level byproducts. This may reflect properties of the annealed oligonucleotide duplexes used as inputs, for example reduced dye binding efficiency or increased heteroduplex formation. Increasing dsDNA input did not restore signal intensity (Fig. S5), suggesting that factors beyond template quantity contributed to the reduced curve quality. A secondary contributor may be an altered local environment in the synthetic UBP-containing template, which contained NaM-5SICS rather than NaM-TPT3. While NaM-TPT3 has been reported to only weakly disrupt the duplex, NaM-5SICS adopts a more intercalating structure that perturbs the local microenvironment, which may further change upon polymerase extension [51,54,55]. Combined with the detrimental energetic properties of UBP-containing heteroduplexes relative to natural mismatches [48], this likely contributes to the leftward shift for the NaM positive control relative to the NaM+ amplicon.

To further validate sequence identities, we employed LC-MS as an established method for UBP detection. Although LC-MS is not inherently quantitative, it can reveal secondary products and thereby provide an indication of amplicon purity. The increased hydrophobicity of UBP-containing products caused a clear chromatographic shift relative to all-natural sequences, which provided a rough indication of UBP incorporation (Figs. S6–7). However, the larger number of peaks detected for UBP-containing products complicated the analysis in two ways. First, the large amounts of input material required (should be in the μg range) resulted in measurements near the detection limit, such that deconvolution of small peaks did not yield confident mass assignments. Second, because deconvolution provides relative intensities within individual peaks (Fig. 6C–E), products eluting in different peaks cannot be quantitatively compared. Together, the positive control and LC-MS analyses support the validity of HRM-based UBP detection, while also highlighting limits in confidence when detecting low-abundance byproducts.

Furthermore, our results highlight several broader challenges and opportunities in UBP amplification. The need for continued optimisation of UBP-incorporating polymerases is underscored by the NaM- control condition, in which natural nucleotides or deletions were readily introduced in the absence of dUNTPs without a reduction in product formation. We observed a preference for substitution by T, consistent with previous reports indicating that the NaM-A mismatch is the most likely to occur [25,31,47,48,56]. Formation of natural-sequence products poses a risk for reliable UBP incorporation, particularly when dUNTP integrity is compromised. Given reported and observed instability of dNaMTP and dTPT3TP under long-term storage or prolonged UV exposure [57,58], HRM may serve as a rapid internal control to monitor dUNTP performance within experimental workflows, showing a shift of the NaM+ difference curve towards the NaM- shape as dUNTP quality declines to indicate that replacement or repurification is needed.

LC-MS analysis additionally revealed nucleotide additions in both natural and UBP-containing amplicons. While the digested, unamplified positive-control samples yielded the expected masses (Fig. S8A–D and Table S3), amplicon fragments displayed a consistent pattern of additions of dA, dG, and dC, and UBP-containing fragments additionally showed apparent incorporation of dNaM or dTPT3, causing a further chromatographic shift of products compared to positive controls (Figs. S6–7 and Table S3). We suspect this reflects the 3′ template-independent nucleotide incorporation activity of Taq polymerase [52], which likely remained active during overnight digestion performed directly after PCR and prior to purification. While 3′ dA addition is well known, incorporation of other natural nucleotides is less efficient, making the origin of the dG and dC additions unclear, although contributions from restriction enzyme processing cannot be excluded. Notably, the 3′ addition activity of the polymerase Dpo4 was previously shown to incorporate a hydrophobic base analogue 100-fold more efficiently than dA [59]. Our data therefore suggest that dNaMTP and dTPT3TP may also be substrates for template-independent 3’ addition by Taq polymerase. This observation warrants further investigation, as it could influence interpretation of non-sequence-specific UBP detection methods, including ours or LC-MS and labelling-based assays.

Consequently, continuous optimisation of UBPs and their corresponding polymerases remains a key challenge for the field [1,11]. HRM analysis may aid these efforts by providing a rapid and accessible screening tool that can be readily integrated into iterative workflow cycles, while also providing insights into unexpected polymerase behaviours. We anticipate that the approach could be extended to additional applications, including RT-PCR-based workflows to assess transcription efficiencies and to support development of UBPs in RNA [14,60]. Building on our positive controls, HRM may be used to compare stability of purified dsDNA products, giving more information on the influence of sequence context on UBP retention and the occurrence and preference for natural-unnatural base mispairing. Combined approaches with existing detection methods, such as controlled UBP-reversion using bridge bases combined with HRM, may further enhance the accuracy and applicability of our method [24,25]. Lastly, the principle demonstrated here may be generalisable to other UBPs and XNAs that alter duplex stability through non-canonical molecular interactions, further extending its impact.

### 4.1 Limitations of the method

Like several existing UBP detection methods, HRM analysis cannot determine sequence identity. Mixtures of base-pair variants yield ambiguous melting patterns and can reveal or exacerbate amplification biases or contamination. Because HRM analysis is inherently comparative, reliable interpretation requires appropriate reference samples, which complicates comparisons when amplification efficiencies differ substantially between samples, as observed for the 2NaM variant. Moreover, highly efficient amplification of short templates increases sensitivity to contamination in no-template controls, necessitating strict separation of pre- and post-PCR workflows and careful reagent handling. This is especially relevant because HRM is not highly quantitative or absolute, and small fractions of secondary products may go unnoticed, which is an important limitation for applications requiring high incorporation fidelity.

Depending on the required sensitivity, HRM should therefore be viewed as a first-line screening approach that provides a reliable qualitative indication of UBP presence or substantial loss, rather than as a replacement for more detailed downstream analyses. Methods such as sequencing or LC-MS may be more appropriate when minute contamination levels or exact sequence identity must be determined. In addition, HRM has been shown to distinguish heterozygous genotypes through heteroduplex formation using an additional melting step after amplification, but incorporating such steps would increase assay complexity. Similarly, detection of strand-switching events (reported for NaM-TPT3) or rare SNPs in sequence contexts that complicate direct resolution may require inclusion of a known genotype as reference [31,50,61]. This would necessitate additional reference samples, thereby increasing assay complexity and potentially complicating downstream purification steps. Sequence context is also known to be an important variable in UBP performance and *in vivo* stability, yet it is currently unclear how different neighbouring bases influence melting behaviour and HRM discrimination. As synthesis of UBP-containing ssDNA becomes more accessible, further systematic study of sequence-context effects will be valuable [56]. Finally, HRM has an inherent limitation related to amplicon length, as reliable analysis generally requires a single melting domain, which is typically only achieved with fragments shorter than 300 bps [35,62]. Because UBP PCR is currently most robust for short amplicons, this constraint does not pose a practical limitation here, but it may become more relevant as UBP amplification protocols are extended to longer targets [12,26].

## 5 Conclusion

Xenobiology remains an emerging field that often requires custom synthesis of chemical components, specialised equipment, and dedicated knowledge. Methodological improvements that expand the experimental toolbox are therefore crucial to accelerate progress and broaden accessibility. For frequent or large-scale screening, which is an increasingly common paradigm in modern biotechnology, speed and cost become decisive factors for R&D processes. Here we present how HRM can be used as a rapid, low-cost verification of UBP incorporation using standard laboratory infrastructure, thereby lowering the threshold for routine experimentation with expanded genetic systems, facilitating broader participation, faster design-build-test-learn cycles, and more systematic assessment of genetic stability and performance. In doing so, it supports the transition of xenobiology from specialised, low-throughput demonstrations toward reproducible, scalable workflows required for translational and industrial applications.

## Author contributions

Conceptualisation and design of experiments: S.V., J.F., B.A., E.A.G. Performance of experiments and data analysis: S.V. Writing - original draft: S.V. Writing - review & editing: S.V., J.F., V.M.d.S., B.A., E.A.G. Funding acquisition: S.V., V.M.d.S., B.A., E.A.G. Resources: B.A. Supervision: J.F., V.M.d.S., B.A., E.A.G. All authors provided critical feedback and helped shape the research, analysis, and manuscript writing.

## Declaration of competing interest

The authors declare that they have no known competing financial interests or personal relationships that could have appeared to influence the work reported in this paper.

## Data Availability

Requests for further information on reported data and resources should be directed to and will be fulfilled by the lead contact, Enrique Asin-Garcia (enrique.asingarcia@wur.nl).
