# Supplementary material for: High-resolution melting analysis for rapid and cost-effective detection of unnatural base pairs
Source: Biodes Res. 2026 May 8;8(2):100090. doi: 10.1016/j.bidere.2026.100090 (PMC13223922; doi:10.1016/j.bidere.2026.100090)
Supplement: Multimedia component 1 [file mmc1.docx]

# Supplementary information

## **Contents**

Table S1: Sequences of oligonucleotides and primers.

Figure S1: Additional data on Figure 2

Figure S2: Additional data on Figure 3

Figure S3: Additional data on Figure 4

Figure S4: Additional data on Figure 5

Table S2: Statistical information on Figure 5C-D

Figure S5: Additional data on Figure 6

Table S3: Overview expected and observed masses LC-MS

Figure S6: HPLC spectra

Figure S7: MS spectra

Figure S8: Deconvoluted MS spectra

***Table S1: Sequences of oligonucleotides and primers used in this study.***

| **Oligonucleotides** | | |
| --- | --- | --- |
| **Sequence (5’-3’)** | **Description** | **Source** |
| aggtctctggagtacaactacaacagccacaacgtcg**t**catcatggccgacaagcagaagaacggcatcaaggtgaacttcaagatccgccacaacatcgaggacggcagcgagagacct | Template sense strand corresponding to ‘T’ | Integrated DNA technologies (IDT) |
| aggtctctcgctgccgtcctcgatgttgtggcggatcttgaagttcaccttgatgccgttcttctgcttgtcggccatgatg**a**cgacgttgtggctgttgtagttgtactccagagacct | Template antisense strand corresponding to ‘T’ | IDT |
| aggtctctggagtacaactacaacagccacaacgtcg**c**catcatggccgacaagcagaagaacggcatcaaggtgaacttcaagatccgccacaacatcgaggacggcagcgagagacct | Template sense strand corresponding to ‘C’ | IDT |
| aggtctctcgctgccgtcctcgatgttgtggcggatcttgaagttcaccttgatgccgttcttctgcttgtcggccatgatg**g**cgacgttgtggctgttgtagttgtactccagagacct | Template antisense strand corresponding to ‘C’ | IDT |
| aggtctctggagtacaactacaacagccacaacgtcgcatcatggccgacaagcagaagaacggcatcaaggtgaacttcaagatccgccacaacatcgaggacggcagcgagagacct | Template sense strand corresponding to ‘del’ | IDT |
| aggtctctcgctgccgtcctcgatgttgtggcggatcttgaagttcaccttgatgccgttcttctgcttgtcggccatgatgcgacgttgtggctgttgtagttgtactccagagacct | Template antisense strand corresponding to ‘del’ | IDT |
| aggtctctggagtacaactacaacagccacaacgtcg**NaM**catcatggccgacaagcagaagaacggcatcaaggtgaacttcaagatccgccacaacatcgaggacggcagcgagagacct | Template sense strand corresponding to ‘NaM’ | ELLA Biotech |
| Aggtctctcgctgccgtcctcgatgttgtggcggatcttgaagttcaccttgatgccgttcttctgcttgtcggccatgatg**5SICS**cgacgttgtggctgttgtagttgtactccagagacct | Template antisense strand corresponding to ‘NaM’ | ELLA Biotech |
| aggtctctggagtacaactacaacagccacaacgtcg**NaM**catcatggccgacaagcagaagaacggcatcaaggtgaacttcaagatccg**NaM**cacaacatcgaggacggcagcgagagacct | Template sense strand corresponding to ‘2NaM’ | ELLA Biotech |
| Aggtctctcgctgccgtcctcgatgttgtg**5SICS**cggatcttgaagttcaccttgatgccgttcttctgcttgtcggccatgatg**5SICS**cgacgttgtggctgttgtagttgtactccagagacct | Template antisense strand corresponding to ‘2NaM’ | ELLA Biotech |
| AGGTCTCTggagtacaactacaacagccac | Forward primer | IDT |
| AGGTCTCTcgctgccgtcctcg | Reverse primer | IDT |


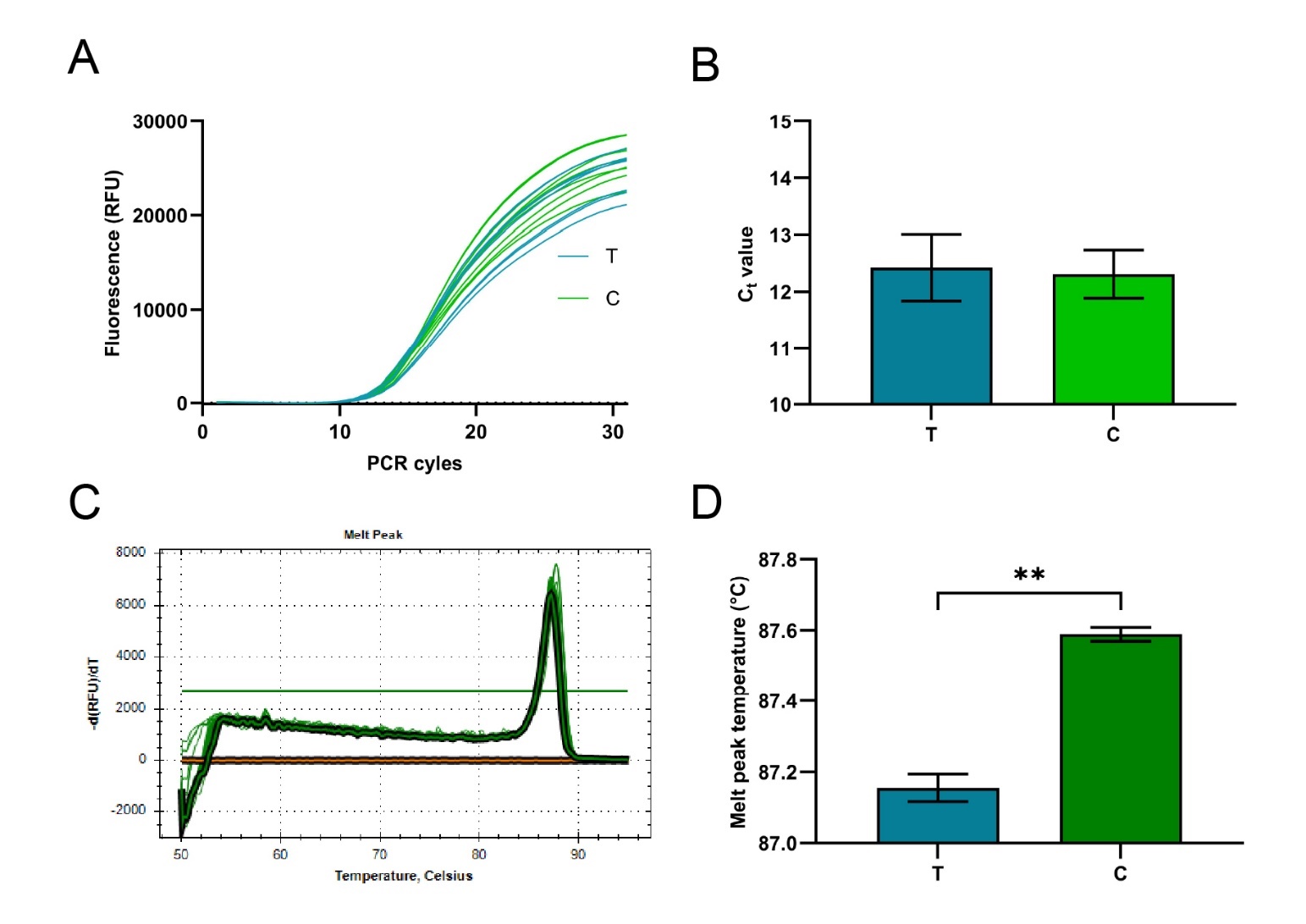


***Figure S1: Additional data on Figure 2 – HRM analysis of T and C sequences****.* ***A)*** *Amplification curves of all replicates from T and C sequences.* ***B)*** *Corresponding Ct values of amplification of T and C as determined by CFX Maestro (errors based on averages of three experiments).* ***C)*** *An exemplary melt peak curve of one of the replicates.* ***D)*** *Melt peak temperatures for T and C as determined by CFX Maestro (errors based on averages of three experiments).*


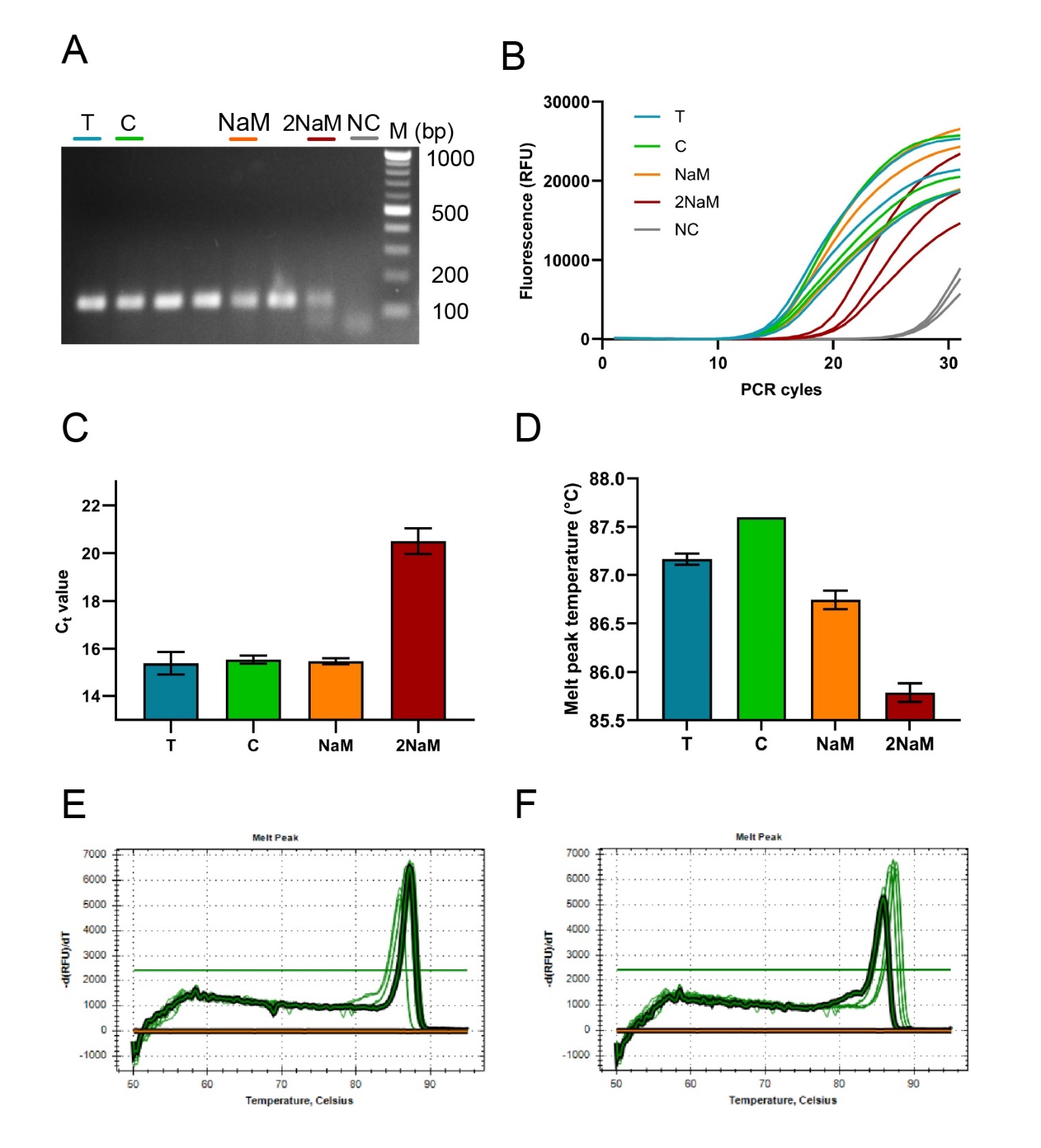
***Figure S2: Additional data on Figure 3 – HRM analysis of T, C, NaM and 2NaM sequences****.* ***A)*** *Gel electrophoresis of amplicons of one replicate.* ***B)*** *Average amplification curves per experiment from all sequences.* ***C)*** *Corresponding Ct values of amplification per experiment (values and baseline determined by CFX Maestro, errors based on averages of three experiments).* ***D)*** *Average melt peak temperatures per experiment (determined by CFX Maestro, errors based on averages of three experiments).* ***E)*** *An exemplary melt peak curve of one replicates of a natural sequence.* ***F)*** *An exemplary melt peak curve of one replicates of a 2NaM sequence.*

***
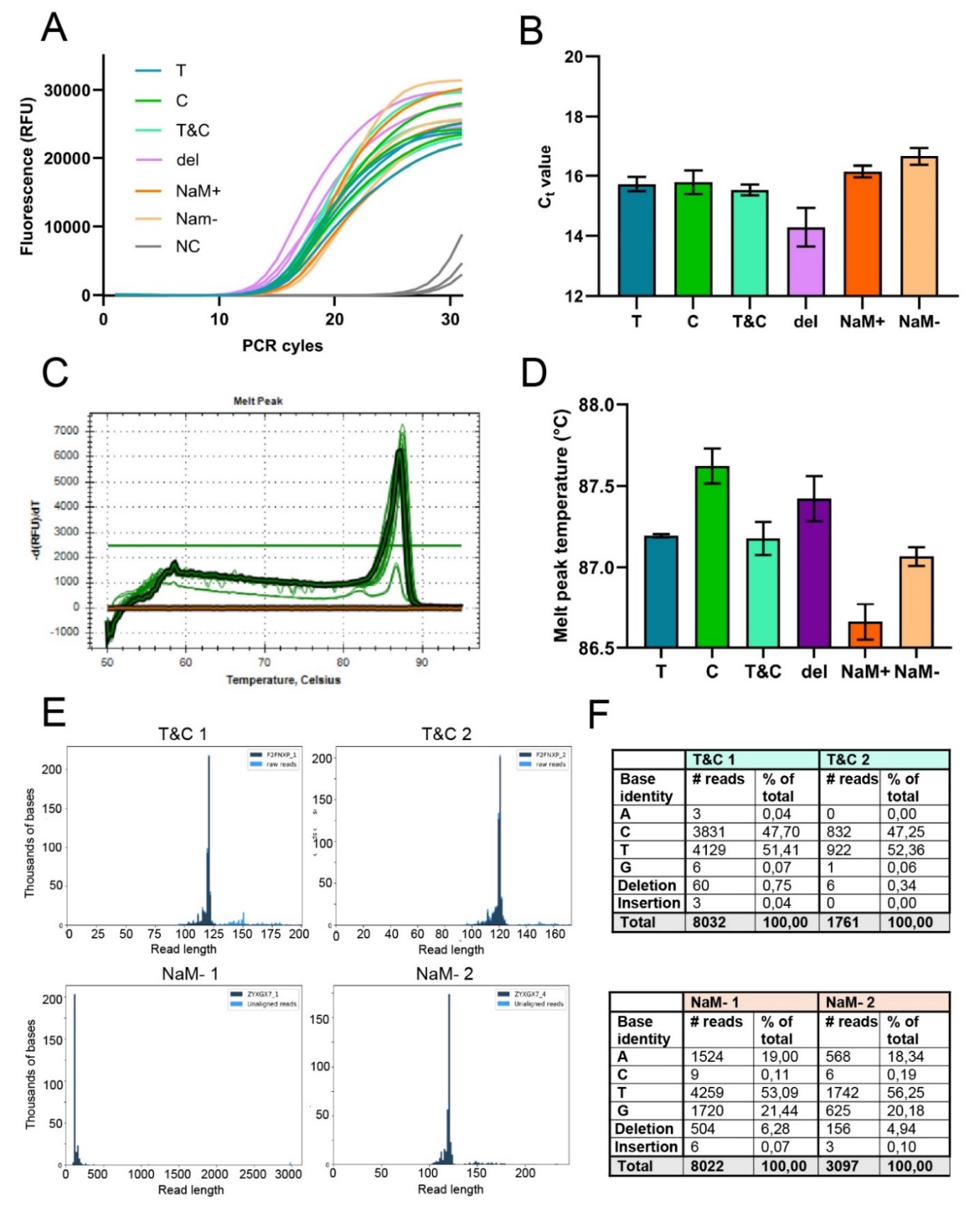
***

***Figure S3: Additional data on Figure 4 – HRM analysis of natural variants, NaM+ and NaM-****.* ***A)*** *Average amplification curves per experiment from all sequences.* ***B)*** *Corresponding Ct values of amplification per experiment (determined by CFX Maestro, errors based on averages of three experiments).* ***C)*** *Exemplary melt peak curve of one replicate of NaM-, showing a slight leftward broadening compared to Figure S2E-F.* ***D)*** *Average melt peak temperatures per experiment (determined by CFX Maestro, errors based on averages of three experiments).* ***E-F)*** *Raw read information of nanopore sequencing of two replicates of variants T&C and NaM- (data provided by Plasmidsaurus).* ***E)*** *Average read lengths.* ***F)*** *Base identity and percentage of overall reads per replicate.*


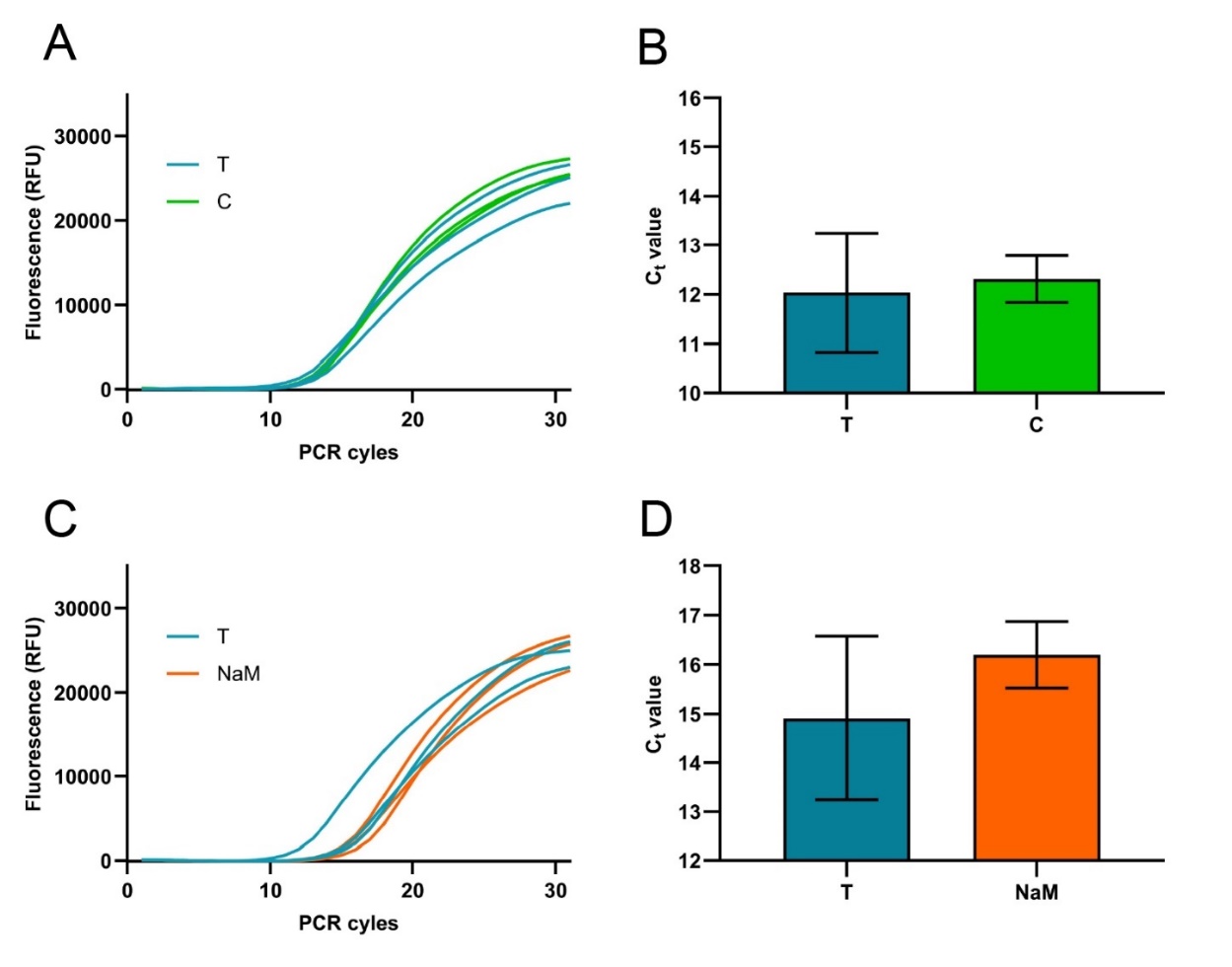


***Figure S4: Additional data on Figure 5 – HRM analysis of mixes of T&C and T&NaM+****.* ***A)*** *Average amplification curves per experiment for T and C mixes.* ***B)*** *Corresponding Ct values of amplification per experiment (values and baseline determined by CFX Maestro, errors based on averages of three experiments).* ***C)*** *Average amplification curves per experiment for T and NaM mixes.* ***D)*** *Corresponding Ct values of amplification per experiment (values and baseline determined by CFX Maestro, errors based on averages of three experiments). NB: one replicate of T showed a lower Ct value, causing a large error bar. However, since amplification proceeded to a similar total fluorescence as the NaM replicate of that experiment (similar product formation confirmed on gel), it was still used for making mixtures.*

***Table S2: Statistical details of one-way ANOVA on T/C and T/NaM mixtures (Figure 5C-D)***

| **T/C ratio** | **Summary** | **Adjusted P Value** |  | **T/NaM ratio** | **Summary** | **Adjusted P Value** |
| --- | --- | --- | --- | --- | --- | --- |
| 100/0 vs. 90/10 | ns | >0,9999 |  | 100/0 vs. 90/10 | ns | 0,9165 |
| 100/0 vs. 75/25 | ns | 0,9989 |  | 100/0 vs. 75/25 | ns | 0,0889 |
| 100/0 vs. 50/50 | ns | 0,6627 |  | 100/0 vs. 50/50 | ** | 0,0036 |
| 100/0 vs. 25/75 | ns | 0,1441 |  | 100/0 vs. 25/75 | **** | <0,0001 |
| 100/0 vs. 10/90 | * | 0,0117 |  | 100/0 vs. 10/90 | ** | 0,0044 |
| 100/0 vs. 0/100 | ** | 0,009 |  | 100/0 vs. 0/100 | * | 0,0108 |
| 90/10 vs. 75/25 | ns | 0,9165 |  | 90/10 vs. 75/25 | ns | 0,1068 |
| 90/10 vs. 50/50 | ns | 0,6627 |  | 90/10 vs. 50/50 | ** | 0,0015 |
| 90/10 vs. 25/75 | ns | 0,1857 |  | 90/10 vs. 25/75 | **** | <0,0001 |
| 90/10 vs. 10/90 | * | 0,0117 |  | 90/10 vs. 10/90 | *** | 0,0009 |
| 90/10 vs. 0/100 | ** | 0,009 |  | 90/10 vs. 0/100 | ** | 0,0075 |
| 75/25 vs. 50/50 | ns | 0,9165 |  | 75/25 vs. 50/50 | * | 0,0361 |
| 75/25 vs. 25/75 | ns | 0,2237 |  | 75/25 vs. 25/75 | * | 0,02 |
| 75/25 vs. 10/90 | * | 0,0268 |  | 75/25 vs. 10/90 | * | 0,0351 |
| 75/25 vs. 0/100 | * | 0,0117 |  | 75/25 vs. 0/100 | * | 0,046 |
| 50/50 vs. 25/75 | ns | 0,0788 |  | 50/50 vs. 25/75 | * | 0,0117 |
| 50/50 vs. 10/90 | * | 0,0389 |  | 50/50 vs. 10/90 | * | 0,0389 |
| 50/50 vs. 0/100 | **** | <0,0001 |  | 50/50 vs. 0/100 | ns | 0,0557 |
| 25/75 vs. 10/90 | ns | 0,2194 |  | 25/75 vs. 10/90 | ns | 0,9165 |
| 25/75 vs. 0/100 | * | 0,0204 |  | 25/75 vs. 0/100 | ns | 0,6627 |
| 10/90 vs. 0/100 | ns | 0,5736 |  | 10/90 vs. 0/100 | ns | 0,5736 |

***
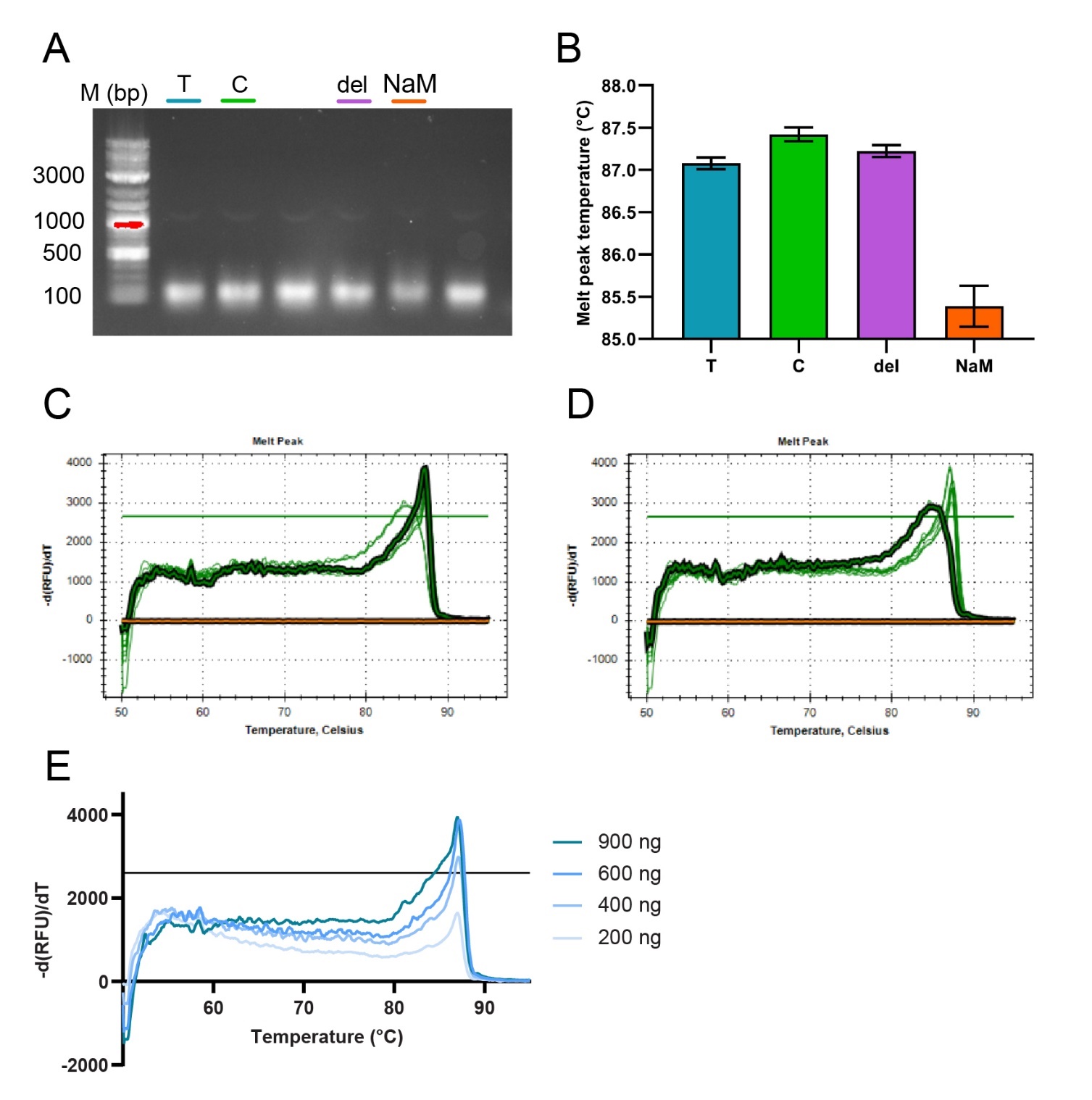
***

***Figure S5: Additional data on Figure 6 – HRM analysis of positive controls of T, C, del and NaM sequences****.* ***A)*** *Gel electrophoresis of dsDNA of one replicate.* ***B)*** *Average melt peak temperatures per experiment (determined by CFX Maestro, errors based on averages of three experiments).* ***C)*** *An exemplary melt peak curve of one replicate of a natural sequence.* ***D)*** *An exemplary melt peak curve of one replicate of a NaM sequence.* ***E)*** *Average melt peak curves of trial experiments testing different oligo concentrations to determine optimal signal intensity (n=2)(horizontal line represents the detection limit as determined by CFX Maestro programme).*

***Table S3: Additional data on LC-MS (Figure 6) – Overview of expected and observed masses of digested templates (positive controls) and HRM samples (PCR amplicons) in Da, corresponding to deconvoluted spectra in Figure S7.*** *PCR products have more expected masses because of the 3’ template-independent activity of Taq polymerase. Mass differences up to 2 Da are assumed to be the same product. In case of a mismatch between expected and observed masses, a putative explanation is provided in the Notes column. Unidentified masses are shown in italics. Masses of putative additions: dA: 313 Da, dT: 304 Da, dG: 329 Da, dC: 289 Da, dTPT3: 345 Da, dNaM: 336 Da, PO_4_-: 79 Da, Na = 23 Da.*

| **Sequence variant** | **Strand** | **Expected (Da)** | **Observed (Da)** | **Notes** |
| --- | --- | --- | --- | --- |
| **Positive controls** | | | | |
| **C** |  |  | 6,654 | Rv primer |
|  |  |  | 9,169 | Fw primer |
|  | Short, antisense | 14,282 | 14,283 |  |
|  | Short, sense | 14,097 | 14,096 |  |
|  | Long, sense | 22,947 | 22,947  22,948 |  |
|  | Long, antisense | 22,737 | 22,736 |  |
| **T&C** |  |  | 6,654 | Rv primer |
|  |  |  | 9,168 | Fw primer |
|  | Short (C), antisense | 14,282 | 14,282 |  |
|  | Short (C), sense | 14,097 | 14,097 |  |
|  | Short (T), antisense | 14,266 | 14,267 |  |
|  | Short (T), sense | 14,112 | 14,111 |  |
|  | Long, sense | 22,947 | 22,947 |  |
|  | Long, antisense | 22,737 | 22,736 |  |
| **del** |  |  | 6,654 | Rv primer |
|  |  |  | 9,168 | Fw primer |
|  | Short, sense | 13,808 | 13,808 |  |
|  | Short, antisense | 13,953 | 13,953 |  |
|  | Long, sense | 22,947 | 22,947 |  |
|  | Long, antisense | 22,737 | 22,736 |  |
| **NaM** |  |  | 6,654 | Rv primer |
|  |  |  | 9,168 | Fw primer |
|  | Short, sense | 14,144 | 14,143 |  |
|  | Short, antisense | 14,306* | *14,289*  *14,292* | *Putative thioamide S ↔ O exchange*  *Contains 5SICS instead of TPT3 |
|  | Long, sense | 22,947 | 22,947 |  |
|  | Long, antisense | 22,737 | 22,735 |  |
| **HRM samples** | | | | |
| **T** | Short, sense | 14,112 | 14,424  14,440  14,441 | + dA  + dG |
|  | Short, antisense | 14,266 | 14,555 | + dC |
|  | Short, antisense + dA | 14,579 | 14,578  14,580  14,581 |  |
|  | Long, sense | 22,947 | 22,644  23,238 | – dT  + dC |
|  | Long, sense +dA | 23,260 | 23,260  23,261 |  |
|  | Long, antisense | 22,737 | 22,735  23,064 | + dG |
| **C** |  |  | *7,754* | *Unknown* |
|  |  |  | *11,631* | *Unknown* |
|  | Short, sense | 14,097 | 14,410  14,425 | + dA  + dG |
|  | Short, antisense | 14,282 | 15,571 | + dC |
|  | Short, antisense + dA | 14,595 | 14,595 |  |
|  | Long, sense | 22,947 | 22,643 | – dT |
|  | Long, sense +dA | 23,260 | 23,260  23,261 |  |
|  | Long, antisense | 22,737 | 22,735  23,064  *22,493* | + dG  *Putative dT nucleoside loss (= 242 Da) due to glycosidic cleavage* |
| **T&C** |  |  | *11,631* | *Unknown* |
|  | Short (T), sense | 14,112 | 14,425 | + dA |
|  | Short (T), antisense | 14,266 |  |  |
|  | Short (T), antisense + dA | 14,579 |  |  |
|  | Short (C), sense | 14,097 | 14,410  14,425 | + dA  + dG |
|  | Short (C), antisense | 14,282 | 15,571 | + dC |
|  | Short (C), antisense + dA | 14,595 | 14,595 |  |
|  | Long, sense | 22,947 | 22,642 | – dT |
|  | Long, sense +dA | 23,260 | 23,261  *23,127* | *Putative loss of A base by depurination (= 135 Da)* |
|  | Long, antisense | 22,737 | 22,735  23,063 | + dG |
|  |  |  | *20,886* | *Putative 6-7 nt truncation* |
| **del** |  |  | *7,754* | *Unknown* |
|  |  |  | *11,631* | *Unknown* |
|  | Short, sense | 13,808 | 14,121  14,136 | + dA  + dG |
|  | Short, antisense | 13,953 | 14,240 | + dC |
|  | Short, antisense + dA | 14,266 | 14,266 |  |
|  | Long, sense | 22,947 | 22,946  22,643 | – dT |
|  | Long, sense +dA | 23,260 | 23,260  23,261 |  |
|  | Long, antisense | 22,737 | 22,737  23,063  *22,490* | + dG  *Putative dT nucleoside loss (= 242 Da) due to glycosidic cleavage* |
| **NaM+** |  |  | *8,975*  *8,978* |  |
|  |  |  | *13,893*  *13,895*  *13,897*  *13,899* | *Putative nucleoside loss from 14,144* |
|  | Short, sense | 14,144 | 14,144  14,457  14,473  14,489 | + dA  + dG  + dTPT3 |
|  | Short, antisense | 14,298 | 14,645  14,634 | + dTPT3  + dNaM |
|  | Short, antisense + dA | 14,611 |  |  |
|  | Long, sense | 22,947 | 23,292  23,282  23,283  23,315 | + dTPT3  + dNaM  + dTPT3 + Na |
|  | Long, sense +dA | 23,260 | 23,261  23,283 | + Na |
|  | Long, antisense | 22,737 | 22,735  22,736  23,062  23,081 | + dG  + dTPT3 |
|  |  |  | *22,769*  *22,518* | *Unknown* |
| **NaM-** |  |  | *7,754* | *Unknown* |
|  |  |  | *8,355* | *Unknown* |
|  |  |  | 14,425 | Corresponds to sequence T (sense) + dA or to C (sense) + dG (see above) |
|  |  |  | 14,441 | Corresponds to sequence T (sense) + dG (see above) |
|  |  |  | 14,555 | Corresponds to sequence G (antisense) + dA or to T (antisense) + dC |
|  |  |  | 14,579 | Corresponds to T (antisense) + dA |
|  |  |  | *18,378*  *18,380*  *18,383* | *Same species, putative 13-15 nt truncation of one of the 74-mers* |
|  | Long, sense | 22,947 | 22,643 | – dT |
|  | Long, sense +dA | 23,260 | 23,261 |  |
|  | Long, antisense | 22,737 | 22,736  23,063 | +dG |
|  |  |  | *23,393* | *Unknown* |

**
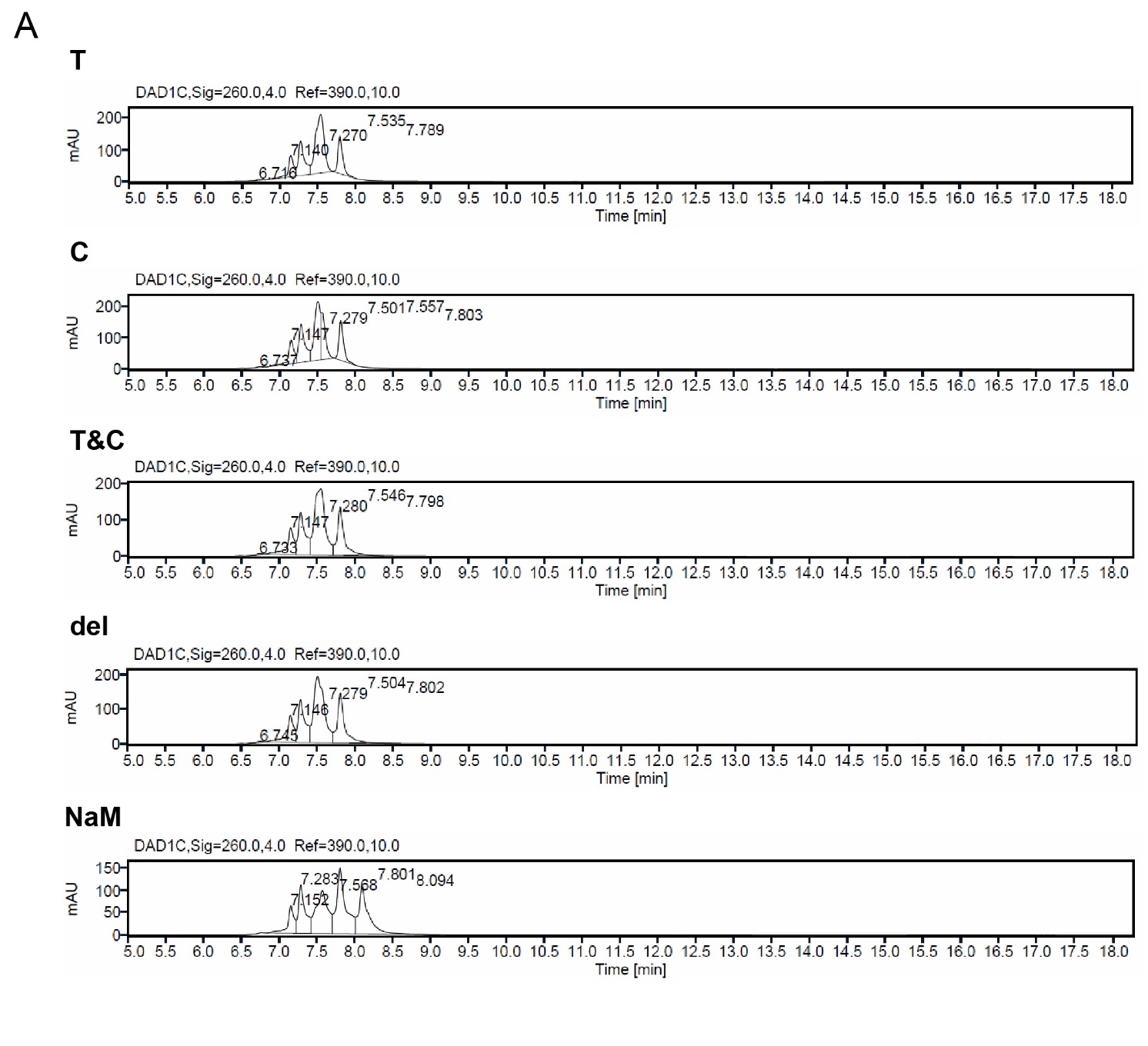
*Figure S6: HPLC traces of dsDNA fragments. A)*** *Positive control samples (continued on next page).*

**
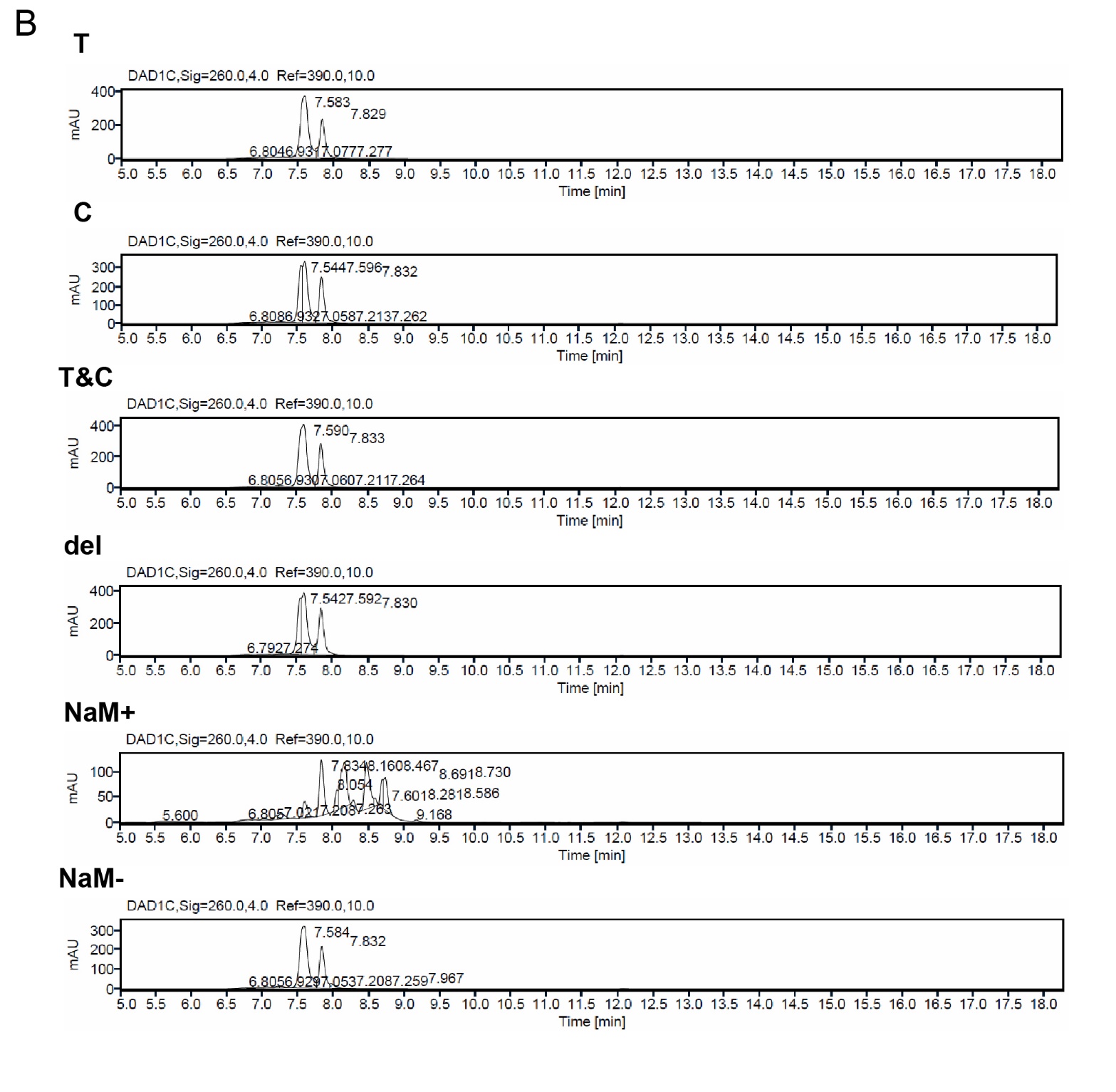
**

***Figure S6: HPLC traces of dsDNA fragments*** *(continued)****. B)*** *HRM PCR amplicons.*

***
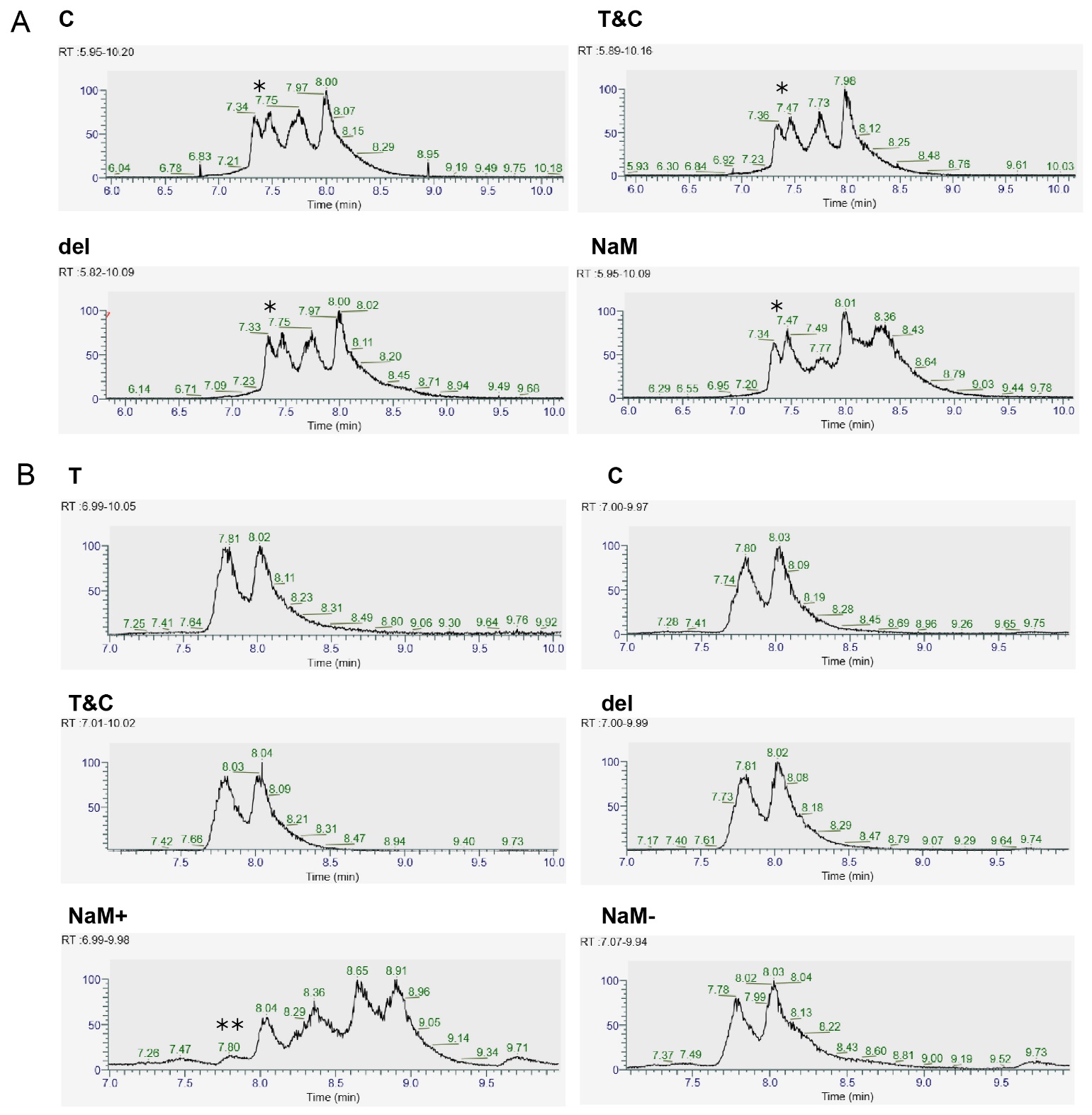
***

***Figure S7: Zoom-in of MS spectra corresponding to HPLC spectra of Figure S6. A)*** *Spectra of positive control samples (sample T could not be obtained due to an injection mistake).* ***B)*** *Spectra of HRM PCR samples. Screensnips made in Freestyle. All peaks shown here were deconvoluted individually, of which results are shown in Figure S8, corresponding to the elution times shown here. * All peaks at 7.25-7.6 min in the positive control samples contained the same two masses of both primers, so only deconvoluted spectra of those peaks from sample C are shown in Figure S8. ** peak intensity was too low for deconvolution.*


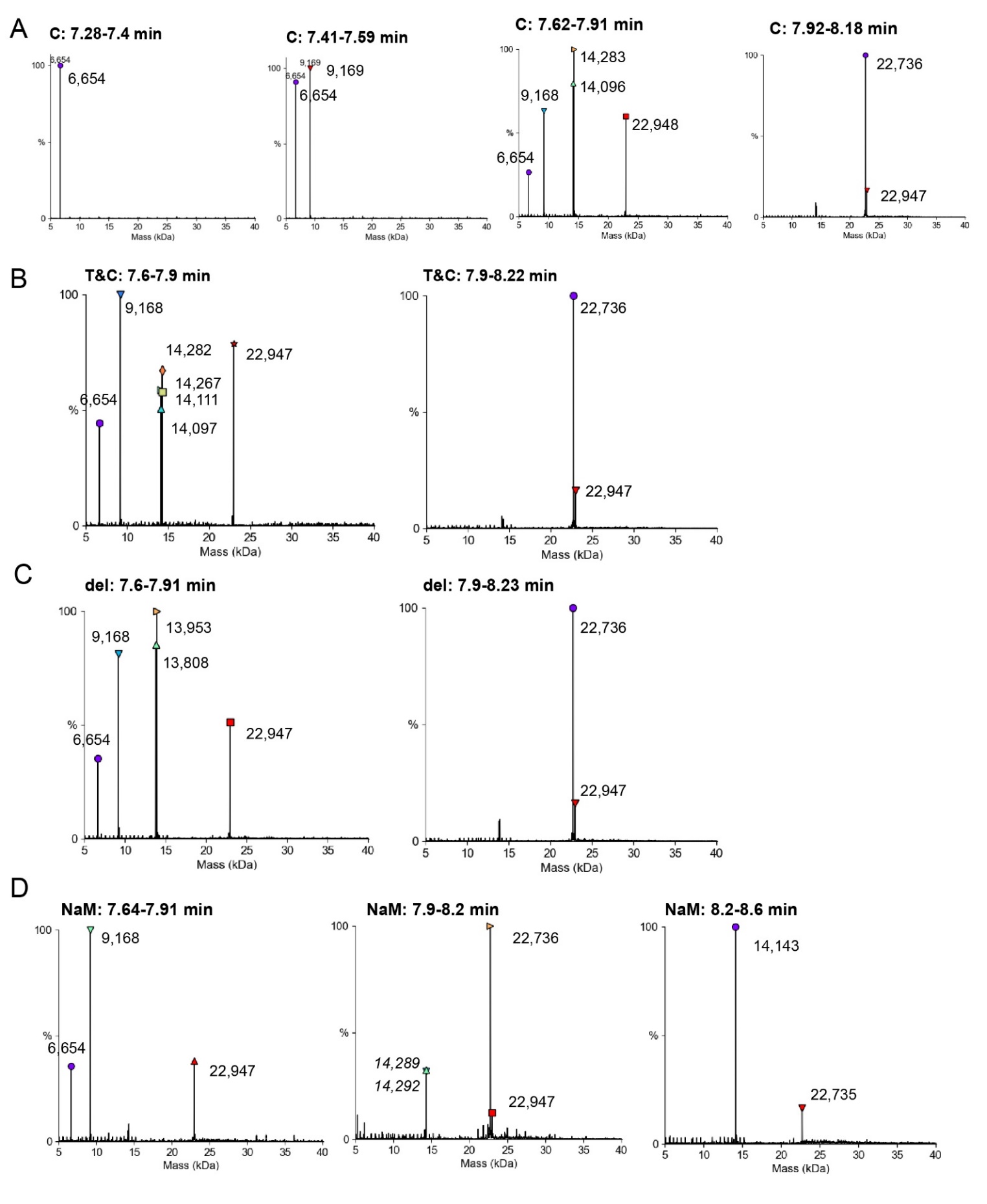
***Figure S8: Complete deconvoluted spectra of digested dsDNA samples*** *(caption continues).* ***A-D)*** *Spectra belonging to positive control samples C (****A****), T&C (****B****), del (****C****) and NaM (****D****).*


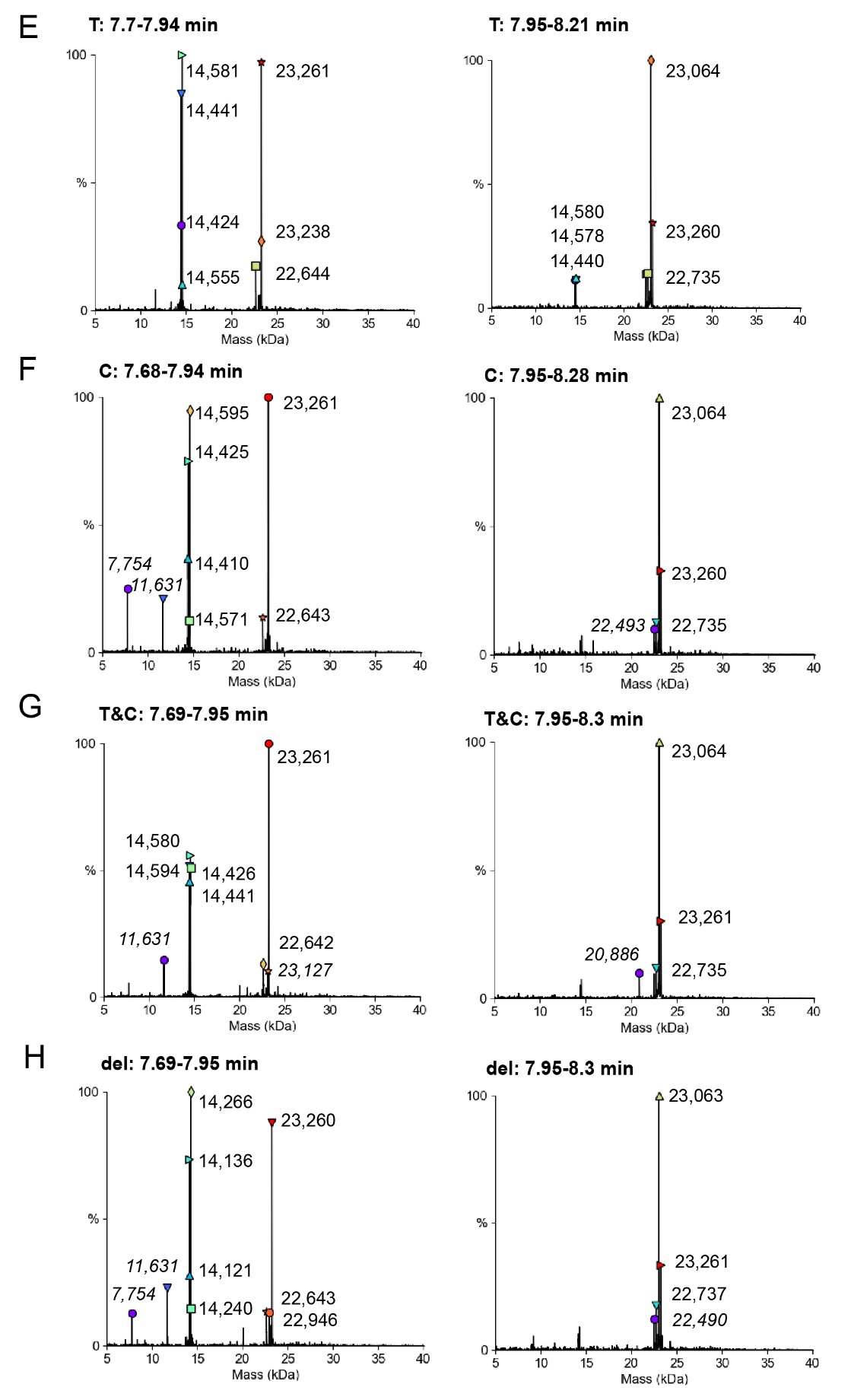
***Figure S8 (continued): Complete deconvoluted spectra of digested dsDNA samples****.* ***E-H****) Spectra belonging to HRM PCR amplicons T (****E****), C (****F****), T&C (****G****), del (****H****).*


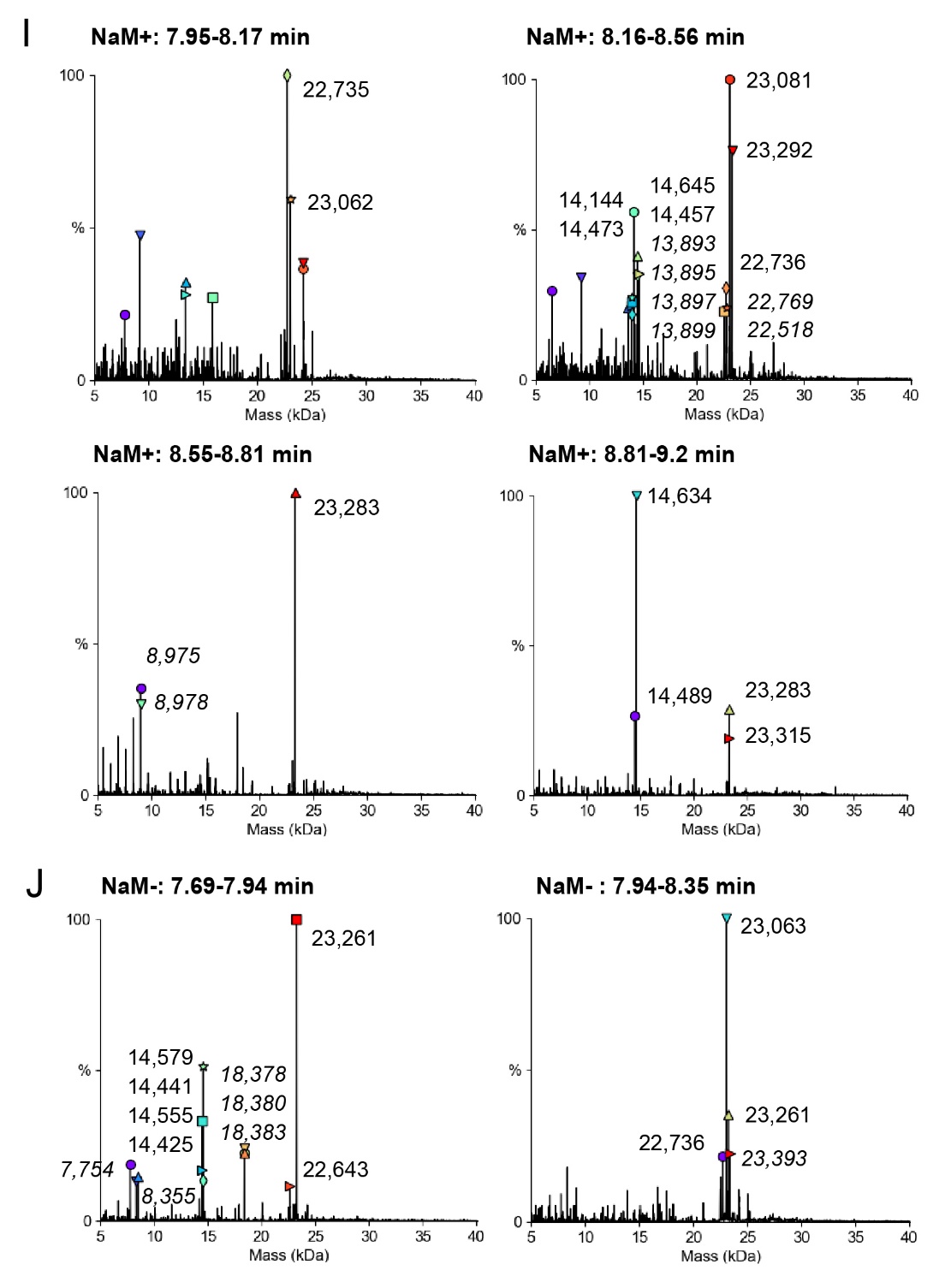


***Figure S8 (continued): Complete deconvoluted spectra of digested dsDNA samples.*** *Each spectrum corresponds to a single integrated peak, indicated by its time stamp (see Figure S7).* ***A-D)*** *Spectra belonging to positive control samples C (****A****), T&C (****B****), del (****C****) and NaM (****D****). Peak threshold value was set to 0.1 for all samples. See Table S4 for assignment of observed masses to expected products.* ***E-J****) Spectra belonging to HRM PCR amplicons T (E), C (F), T&C (G), del (H), NaM+ (I) and NaM- (J). Peak threshold values were set to 0.1 for natural template samples and 0.2 for unnatural templates. See Table S4 for assignment of observed masses to expected products (masses shown in italics are putative). The first peak of NaM+ (7.7-7.9 min) could not be deconvoluted and is not shown (as indicated by ** in Figure S7).*
